# Supplementary material for: Whole genome sequencing of Neisseria meningitidis Y isolates collected in the Czech Republic in 1993-2018
Source: PLoS One. 2022 Mar 10;17(3):e0265066. doi: 10.1371/journal.pone.0265066 (PMC8912901; doi:10.1371/journal.pone.0265066)
Supplement: S1 Fig — (PDF) [file pone.0265066.s001.pdf]

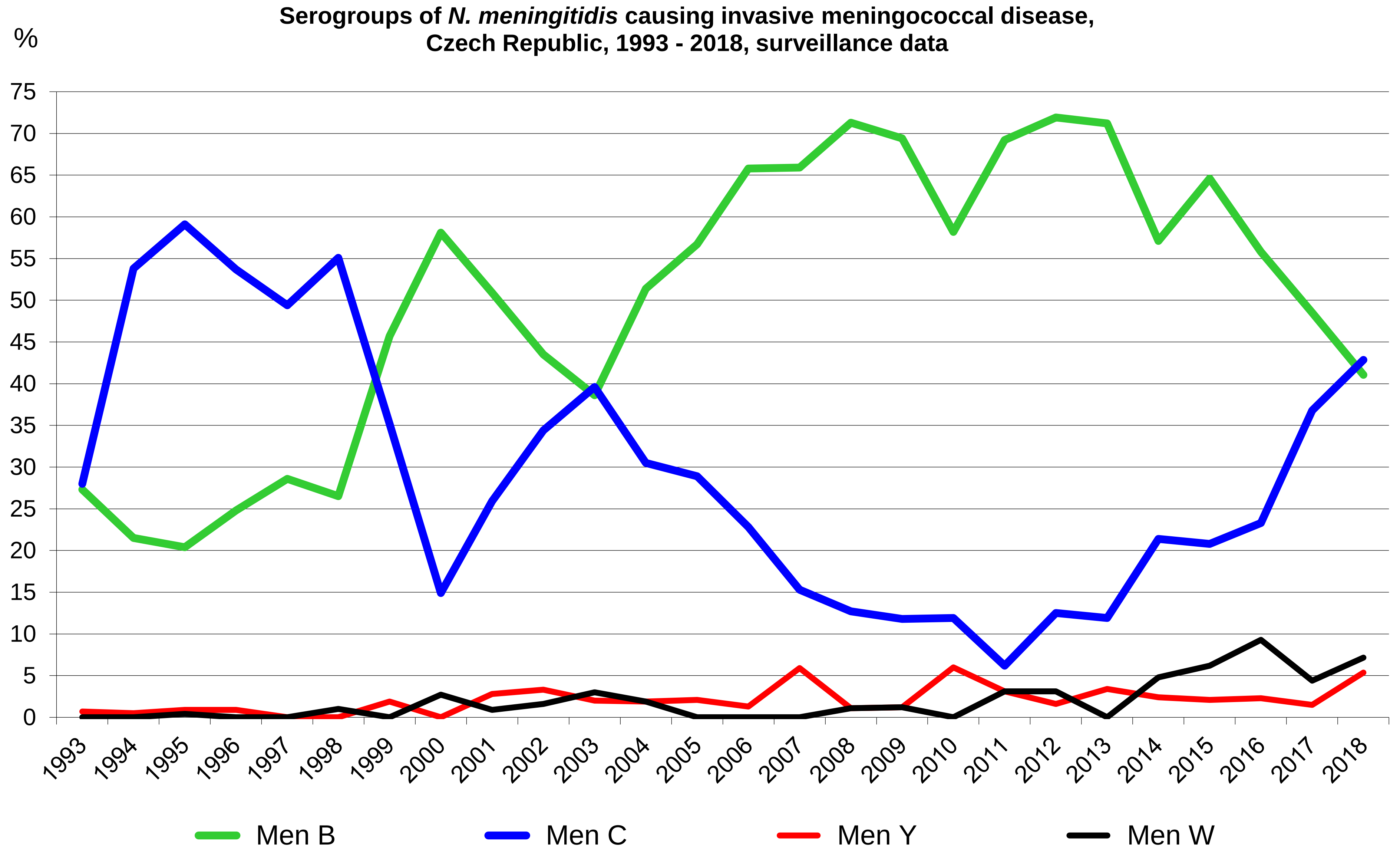

Serogroups of *N. meningitidis* causing invasive meningococcal disease,
Czech Republic, 1993 - 2018, surveillance data
%
75
70
65
60
55
50
45
40
35
30
25
20
15
10
5
0
1993
1994
1995
1996
1997
1998
1999
2000
2001
2002
2003
2004
2005
2006
2007
2008
2009
2010
2011
2012
2013
2014
2015
2016
2017
2018
Men B
Men C
Men Y
Men W
