# Supplementary material for: Whole genome sequencing of Neisseria meningitidis Y isolates collected in the Czech Republic in 1993-2018
Source: PLoS One. 2022 Mar 10;17(3):e0265066. doi: 10.1371/journal.pone.0265066 (PMC8912901; doi:10.1371/journal.pone.0265066)
Supplement: S2 Fig — (PDF) [file pone.0265066.s002.pdf]

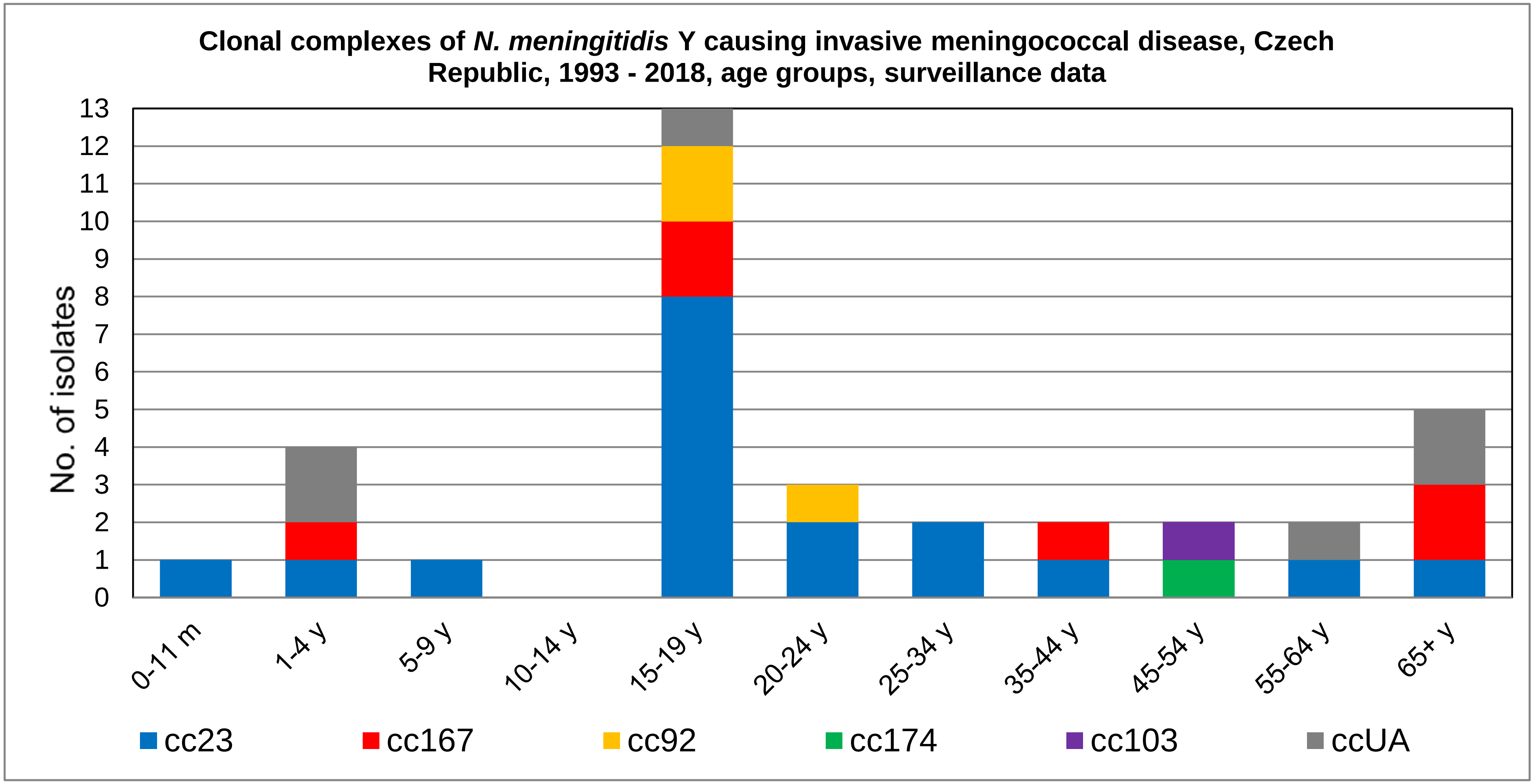
Clonal complexes of *N. meningitidis* Y causing invasive meningococcal disease, Czech Republic, 1993 - 2018, age groups, surveillance data
No. of isolates
0
1
2
3
4
5
6
7
8
9
10
11
12
13
0-11 m
1-4 y
5-9 y
10-14 y
15-19 y
20-24 y
25-34 y
35-44 y
45-54 y
55-64 y
65+ y
cc23
cc167
cc92
cc174
cc103
ccUA
