# Supplementary material for: Whole genome sequencing of Neisseria meningitidis Y isolates collected in the Czech Republic in 1993-2018
Source: PLoS One. 2022 Mar 10;17(3):e0265066. doi: 10.1371/journal.pone.0265066 (PMC8912901; doi:10.1371/journal.pone.0265066)
Supplement: S3 Fig — (PDF) [file pone.0265066.s003.pdf]

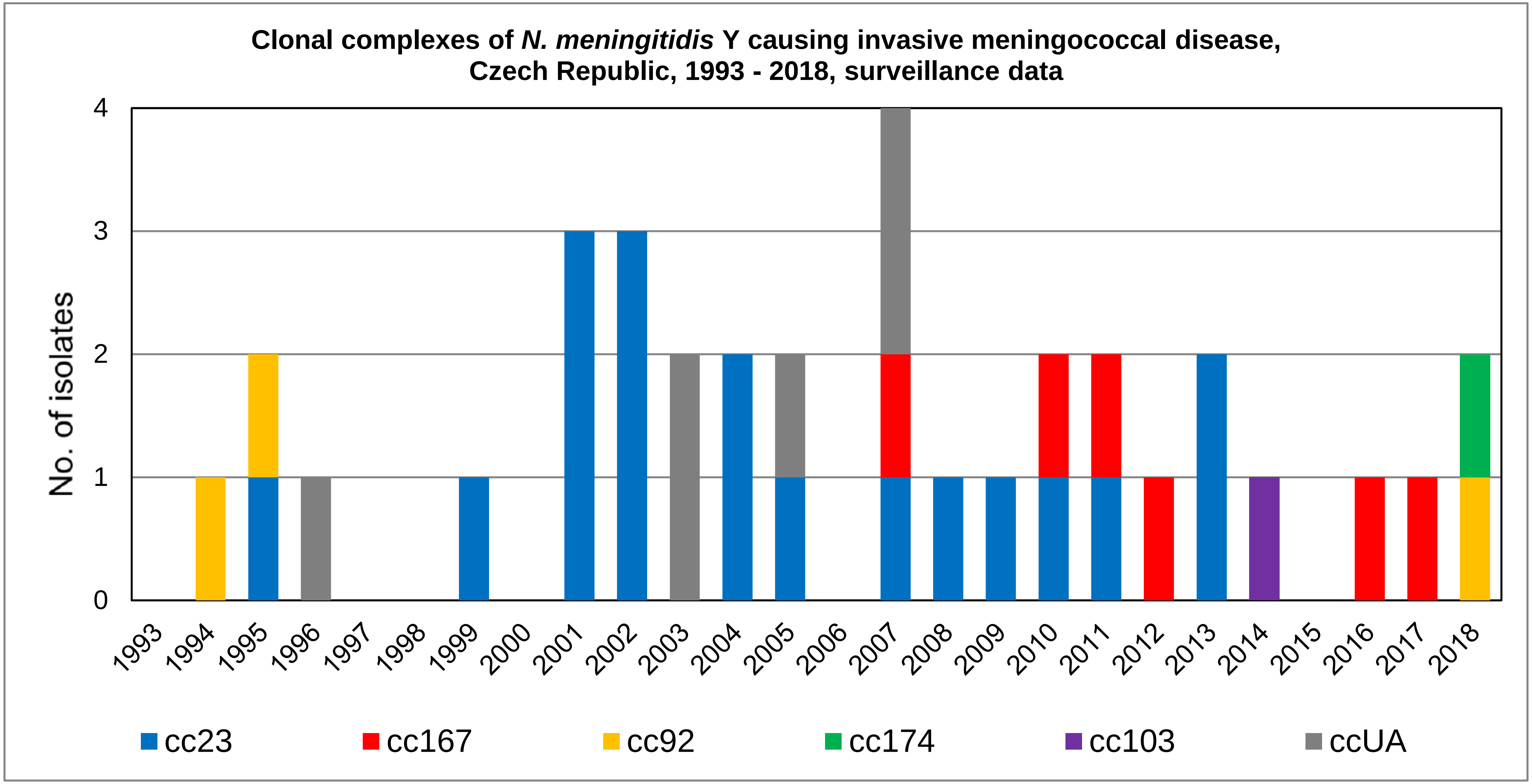

Clonal complexes of N. meningitidis Y causing invasive meningococcal disease,
Czech Republic, 1993 - 2018, surveillance data
No. of isolates
0
1
2
3
4
1993
1994
1995
1996
1997
1998
1999
2000
2001
2002
2003
2004
2005
2006
2007
2008
2009
2010
2011
2012
2013
2014
2015
2016
2017
2018
cc23
cc167
cc92
cc174
cc103
ccUA
