# Supplementary material for: Whole genome sequencing of Neisseria meningitidis Y isolates collected in the Czech Republic in 1993-2018
Source: PLoS One. 2022 Mar 10;17(3):e0265066. doi: 10.1371/journal.pone.0265066 (PMC8912901; doi:10.1371/journal.pone.0265066)
Supplement: Attachment — Submitted filename: PONE-D-21-29616_edits for authors.pdf [file pone.0265066.s004.pdf]

PLOS ONE

Whole Genome Sequencing of Neisseria meningitidis Y isolates collected in the Czech Republic in 1993-2018

--Manuscript Draft--

**Manuscript Number:**	PONE-D-21-29616
**Article Type:**	Research Article
**Full Title:**	Whole Genome Sequencing of Neisseria meningitidis Y isolates collected in the Czech Republic in 1993-2018
**Short Title:**	Whole Genome Sequencing of Neisseria meningitidis Y, Czech Republic, 1993-2018
**Corresponding Author:**	Pavla Krizova, M.D., Ph.D.<br>NIPH: Statni Zdravotni Ustav<br>Prague, CZECH REPUBLIC
**Keywords:**	Neisseria meningitidis, serogroup Y, whole genome sequencing
**Abstract:**	Introduction<br>The study presents the analysis of whole genome sequencing (WGS) data for Neisseria meningitidis serogroup Y isolates collected in the Czech Republic in the period 1993-2018 and their comparison to other countries.<br><br>Material and methods<br>The WGS data of 43 Czech N. meningitidis Y isolates, 35 from invasive meningococcal disease and 8 from healthy carriers were analysed. The WGS method used in the present study has been described in detail previously.<br><br>Results<br>WGS assigned 36 isolates of N. meningitidis Y to five clonal complexes: cc23, cc92, cc167, cc103, and cc174, while seven isolates remained unassigned to any clonal complexes (ccUA). Eighteen invasive isolates belonged to clonal complex cc23, which was detected throughout the studied years. The occurrence of cc23 was recorded in all age groups of IMD patients, being the highest in 15-19-year-olds. The Czech invasive isolates of N. meningitidis Y can be considered as a heterogeneous population, about two thirds of which are genetically closely related and belong to cc23. The remaining isolates are assigned to a number of other clonal complexes and show a very low relatedness to cc23 isolates and to each other. The Czech isolates of N. meningitidis Y generally follow the trend observed for European isolates. The Czech isolates of N. meningitidis Y, added to the non-European isolates for comparison, do not follow the observed non-European trend. Their high clonal heterogeneity considerably contributes to the overall heterogeneity of the phylogenetic network, which contains Czech and non-European isolates.<br><br>Conclusions<br>The most represented clonal complex in Czech N. meningitidis Y isolates is cc23, which forms a separate lineage, distinct from all other isolates of N. meningitidis Y. The WGS analysis showed the population of Czech N. meningitidis Y isolates as very heterogeneous, containing a large number of unrelated genetic lineages.
**Order of Authors:**	Michal Honskus
	Zuzana Okonji
	Martin Musilek
	Pavla Krizova, M.D., Ph.D.
**Opposed Reviewers:**	
**Additional Information:**	
**Question**	**Response**
**Financial Disclosure**	Supported by Ministry of Health of the Czech Republic, grant no. NV19-09-00319. All

Powered by Editorial Manager® and ProduXion Manager® from Aries Systems Corporation

Enter a financial disclosure statement that describes the sources of funding for the work included in this submission. Review the submission guidelines for detailed requirements. View published research articles from *PLOS ONE* for specific examples.<br><br>This statement is required for submission and **will appear in the published article** if the submission is accepted. Please make sure it is accurate.<br><br>**Unfunded studies**<br>Enter: *The author(s) received no specific funding for this work.*<br><br>**Funded studies**<br>Enter a statement with the following details:<br>• Initials of the authors who received each award<br>• Grant numbers awarded to each author<br>• The full name of each funder<br>• URL of each funder website<br>• Did the sponsors or funders play any role in the study design, data collection and analysis, decision to publish, or preparation of the manuscript?<br>• **NO** - Include this sentence at the end of your statement: *The funders had no role in study design, data collection and analysis, decision to publish, or preparation of the manuscript.*<br>• **YES** - Specify the role(s) played.<br><br>* typeset	rights<br>reserved. The funding had no role in study design, data collection and analysis, decision to publish, or preparation of the manuscript.
**Competing Interests**<br><br>Use the instructions below to enter a competing interest statement for this submission. On behalf of all authors, disclose any competing interests that could be perceived to bias this work—acknowledging all financial support and any other relevant financial or non-financial competing interests.<br><br>This statement is required for submission and **will appear in the published article** if	The authors have declared that no competing interests exist. This does not alter our adherence to PLOS ONE policies on sharing data and materials.

the submission is accepted. Please make sure it is accurate and that any funding sources listed in your Funding Information later in the submission form are also declared in your Financial Disclosure statement.<br><br>View published research articles from *PLOS ONE* for specific examples.<br><br>**NO authors have competing interests**<br><br>Enter: *The authors have declared that no competing interests exist.*<br><br>**Authors with competing interests**<br><br>Enter competing interest details beginning with this statement:<br><br>*I have read the journal's policy and the authors of this manuscript have the following competing interests: [insert competing interests here]*<br><br>* typeset	
**Ethics Statement**<br><br>Enter an ethics statement for this submission. This statement is required if the study involved:<br><br>• Human participants<br>• Human specimens or tissue<br>• Vertebrate animals or cephalopods<br>• Vertebrate embryos or tissues<br>• Field research<br><br>Write "N/A" if the submission does not require an ethics statement.<br><br>General guidance is provided below. Consult the submission guidelines for detailed instructions. **Make sure that all information entered here is included in the Methods section of the manuscript.**	N/A

Powered by Editorial Manager® and ProduXion Manager® from Aries Systems Corporation

**Format for specific study types**<br><br>**Human Subject Research (involving human participants and/or tissue)**<br>• Give the name of the institutional review board or ethics committee that approved the study<br>• Include the approval number and/or a statement indicating approval of this research<br>• Indicate the form of consent obtained (written/oral) or the reason that consent was not obtained (e.g. the data were analyzed anonymously)<br><br>**Animal Research (involving vertebrate animals, embryos or tissues)**<br>• Provide the name of the Institutional Animal Care and Use Committee (IACUC) or other relevant ethics board that reviewed the study protocol, and indicate whether they approved this research or granted a formal waiver of ethical approval<br>• Include an approval number if one was obtained<br>• If the study involved *non-human primates*, add *additional details* about animal welfare and steps taken to ameliorate suffering<br>• If anesthesia, euthanasia, or any kind of animal sacrifice is part of the study, include briefly which substances and/or methods were applied<br><br>**Field Research**<br><br>Include the following details if this study involves the collection of plant, animal, or other materials from a natural setting:<br>• Field permit number<br>• Name of the institution or relevant body that granted permission	
**Data Availability**<br><br>Authors are required to make all data underlying the findings described fully available, without restriction, and from the time of publication. PLOS allows rare exceptions to address legal and ethical concerns. See the PLOS Data Policy and FAQ for detailed information.	Yes - all data are fully available without restriction

Powered by Editorial Manager® and ProduXion Manager® from Aries Systems Corporation

A Data Availability Statement describing where the data can be found is required at submission. Your answers to this question constitute the Data Availability Statement and **will be published in the article**, if accepted.<br><br>**Important:** Stating 'data available on request from the author' is not sufficient. If your data are only available upon request, select 'No' for the first question and explain your exceptional situation in the text box.<br><br>Do the authors confirm that all data underlying the findings described in their manuscript are fully available without restriction?	
**Describe where the data may be found in full sentences. If you are copying our sample text, replace any instances of XXX with the appropriate details.**<br><br>• If the data are **held or will be held in a public repository**, include URLs, accession numbers or DOIs. If this information will only be available after acceptance, indicate this by ticking the box below. For example: *All XXX files are available from the XXX database (accession number(s) XXX, XXX.).*<br>• If the data are all contained **within the manuscript and/or Supporting Information files**, enter the following: *All relevant data are within the manuscript and its Supporting Information files.*<br>• If neither of these applies but you are able to provide **details of access elsewhere**, with or without limitations, please do so. For example:<br><br>*Data cannot be shared publicly because of [XXX]. Data are available from the XXX Institutional Data Access / Ethics Committee (contact via XXX) for researchers who meet the criteria for access to confidential data.*<br><br>*The data underlying the results presented in the study are available from (include the name of the third party*	All WGS data are available on webpage of the Neisseria PubMLST database (www.pubmlst.org/neisseria/), under the ID presented in Table 1 of the Manuscript.

and contact information or URL).<br>• This text is appropriate if the data are owned by a third party and authors do not have permission to share the data.<br><br>* typeset	
Additional data availability information:	

Powered by Editorial Manager® and ProduXion Manager® from Aries Systems Corporation

NATIONAL INSTITUTE OF PUBLIC HEALTH
Šrobárova 48
Praha 10
100 42
Czech Republic

Editorial Board
PlosOne

Prague, September 13, 2021

Dears,

On behalf of authors, I submit a manuscript „**Whole Genome Sequencing of *Neisseria meningitidis* Y isolates collected in the Czech Republic in 1993-2018".**

This research article presents the analysis of WGS data for Czech *Neisseria meningitidis* serogroup Y isolates and their comparison with WGS data of *Neisseria meningitidis* serogroup Y from other countries.

The manuscript has not been published previously or submitted for consideration for publication elsewhere. All authors have contributed to the manuscript and approved the final version of the manuscript.

I am responsible for submission, corresponding and proofs.

Yours sincerely,

Pavla Krizova, M.D., Ph.D.
Head, National Reference Laboratory for Meningococcal Infections
Center for Epidemiology and Microbiology
National Institute of Public Health
Srobarova 49/48
100 00 Prague 10
Czech Republic

Manuscript
Click here to access/download;Manuscript;27-WGS-N.m.Y.docx

Whole Genome Sequencing of *Neisseria meningitidis* Y isolates collected in the Czech Republic in 1993-2018

Michal Honskus, Zuzana Okonji, Martin Musilek and Pavla Krizova*

National Reference Laboratory for Meningococcal Infections, Centre for Epidemiology and Microbiology, National Institute of Public Health, Prague, Czech Republic

*Corresponding author
E-mail: pavla.krizova@szu.cz (PK)

Abstract

Introduction

The study presents the analysis of whole genome sequencing (WGS) data for *Neisseria meningitidis* serogroup Y isolates collected in the Czech Republic ~~in the period 1993-2018~~ and their comparison to other countries.

Material and methods

The WGS data of 43 Czech *N. meningitidis* Y isolates, 35 from invasive meningococcal disease and 8 from healthy carriers were analysed. The WGS method used in the present study has been described in detail previously.

Results

WGS assigned 36 isolates of *N. meningitidis* Y to five clonal complexes: cc23, cc92, cc167, cc103, and cc174, while seven isolates remained unassigned to any clonal complexes (ccUA). Eighteen invasive isolates belonged to clonal complex cc23, which was detected throughout the studied years. The occurrence of cc23 was recorded in all age groups of IMD patients, being the highest in 15-19-year-olds. The Czech invasive isolates of *N. meningitidis* Y can be considered as a heterogeneous population, about two thirds of which are genetically closely related and belong to cc23. The remaining isolates are assigned to a number of other clonal complexes and ~~show a~~ very low relatedness to cc23 isolates and to each other. The Czech isolates of *N. meningitidis* Y generally follow the trend observed for European isolates. ~~The Czech isolates of *N. meningitidis* Y, added to the~~ non-European isolates ~~for comparison,~~ do not follow the observed non-European trend. Their high clonal heterogeneity considerably contributes to the overall heterogeneity of the phylogenetic network, which contains Czech and non-European isolates.

Conclusions

The most represented clonal complex in Czech *N. meningitidis* Y isolates is cc23, which forms a separate lineage, distinct from all other isolates of *N. meningitidis* Y. The WGS analysis showed the population of Czech *N. meningitidis* Y isolates as very heterogeneous, containing a large number of unrelated genetic lineages.

Introduction

Invasive meningococcal disease (IMD) is one of the most severe infectious diseases worldwide with a high fatality rate and a high risk of life-long disabling sequelae in survivors [1, 2, 3].

IMD is caused by the bacterium *Neisseria meningitidis*, detected in the upper respiratory tract of up to 10 % of the healthy population, referred to as healthy carriers of meningococci. The serogroups of *N. meningitidis* most often involved in IMD are A, B, C, X, W, and Y. Their distribution varies between countries [4, 5, 6].

The incidence of IMD and causative serogroups vary over time due to a combination of effects of vaccination programmes. After meningococcal C conjugate vaccines were introduced in the early 2000s, a sharp decline in IMD caused by *N. meningitidis* serogroup C was observed in a number of countries. When becoming available in the African meningitis belt, meningococcal A conjugate vaccine practically led to the elimination of IMD caused by *N. meningitidis* serogroup A in this area [3]. Consequently, other causative serogroups started to be dominant. Increasing trends were observed particularly for serogroups W and Y, which resulted in switching from monovalent meningococcal C conjugate vaccines to quadrivalent meningococcal A, C, W, Y conjugate vaccines in many countries [3].

Since the 1990s IMD caused by serogroup Y showed an upward trend in the USA, Canada, Latin American countries, but also in Europe, particularly in Sweden [6, 7, 8, 9, 10, 11, 12, 13, 14]. Before the worldwide rise in IMD caused by serogroup Y, this serogroup was detected primarily in healthy carriers, being rarely the cause of IMD and if so, particularly in

the elderly population. The recent rise in IMD cases caused by serogroup Y has also been reported in younger age groups.

Molecular characterization of isolates of *N. meningitidis* Y has shown that the recent rise in IMD caused by this serogroup is primarily linked to clonal complex cc23, which, however, had a relatively low invasive potential compared to other hyperinvasive lineages [11]. The study from 2014 of Swedish IMD *N. meningitidis* Y isolates by whole genome sequencing (WGS) has shown that this increase in IMD was due to the spread of some more virulent variants of the cc23 clonal complex [11].

In the Czech Republic, data on the incidence of IMD have been available since 1943 (passive reporting) along with surveillance data since 1993 [15]. The surveillance data-based analysis of the epidemiological situation of IMD in the Czech Republic and the meningococcal vaccination guidelines are published annually in the Bulletin of the Centre for Epidemiology and Microbiology (http://www.szu.cz/publikace/zpravy-epidemiologie-a-mikrobiologie). The IMD surveillance data indicate that serogroups B and C are dominant in the Czech Republic (ranging from 20.4 to 71.9 % and from 6.6 to 59.2 %, respectively), but since the early millennium, serogroups W and Y have been on the rise: up to 9.3 % and 5.9 %, respectively (S1 Fig). The IMD surveillance programme in the Czech Republic also includes molecular characterization of isolates performed by the National Reference Laboratory for Meningococcal Infections (NRL) [15], which has been extended by the WGS method since 2015 [16, 17, 18].

Vaccines registered by the European Medicines Agency (EMA) are available in the Czech Republic for vaccination against IMD – two quadrivalent conjugate vaccines containing

antigens of four meningococcal serogroups, A, C, W, and Y: MenACWY-TT vaccine (Nimenrix) and MenACWY-CRM vaccine (Menveo) and two recombinant vaccines including serogroup B meningococcal antigens (MenB vaccines): MenB-4C vaccine (Bexsero) and MenB-fHbp vaccine (Trumenba). Since 2014, the Czech Vaccinology Society of the J. E. Purkyně Czech Medical Association in cooperation with the NRL draws up meningococcal vaccination recommendations, which are continuously updated. To achieve the most comprehensive possible immunity against IMD, the combination of a quadrivalent ACWY conjugate vaccine and MenB vaccine is recommended in the Czech Republic. The update of the Recommendation on vaccination against IMD is available on the websites of the Czech Vaccinology Society (https://www.vakcinace.eu/doporuceni-a-stanoviska) and of the NRL (http://www.szu.cz/ockovani-proti-meningokokovym-onemocnenim), providing detailed information on the vaccination of infants and small children, adolescents and young adults, and high-risk groups. In accordance with Czech legislation, vaccination against IMD is covered by health insurance for persons with underlying diseases (since January 2018) and small children (since May 2020). The recommendation of the Czech Vaccinology Society of the J. E. Purkyně Czech Medical Association on integrating meningococcal vaccines (quadrivalent ACWY conjugate vaccine and MenB vaccine) in the adolescent immunisation programme, preferably before the age of 15, has not yet been implemented – legislative approval of this recommendation is currently expected.

Since 1993, the overall case fatality rate of IMD in the Czech Republic has been ranging from 4.7 to 16.4 %, averaging at 10.2 %. The highest case fatality rate is due to IMD caused by *N. meningitidis* Y averaging at 15.5 %. Molecular characterization of *N. meningitidis* Y isolates provides the necessary background data for setting the vaccination strategy in the Czech

Republic. We present results of the WGS analysis of 43 Czech isolates of *N. meningitidis* Y from 1993-2018 and their comparison with the WGS data from other countries.

Material and methods

Neisseria meningitidis isolates

Isolates of *N. meningitidis* from IMD patients and healthy carriers are referred to the NRL by microbiological laboratories from all over the Czech Republic for confirmation and further characterization in accordance with the Czech legislation. These isolates are stored freeze-dried and/or frozen (-80 ^{o}C, Cryobank B, ITEST) in the NRL collection. For each isolate, clinical, epidemiological, and microbiological data are available in the electronic database of the NRL collection. 43 isolates *N. meningitidis* Y from 1993-2018 were studied (35 from IMD patient and 8 from healthy carriers).

Identification and characterization of *Neisseria meningitidis*

N. meningitidis isolates were cultured on Mueller Hinton chocolate agar at 37 ^{o}C and 5% CO_2 for 18-24 hours. The identification of *N. meningitidis* was confirmed using the API NH kit (BIOMÉRIEUX). Serogroups were determined by conventional serological methods (Pastorex Meningitis Bio-RAD, antisera *N. meningitidis* ITEST, Bio-RAD) and confirmed by RT-PCR.

DNA extraction, WGS protocol, and WGS data analysis and visualisation

The methods used in the present study have been described in detail previously [17, 18]. DNA isolation was performed using the QIAamp DNA Mini Kit (QIAGEN) according to the manufacturer's instructions. DNA was sent for WGS analysis to the European Molecular Biology Laboratory, Heidelberg, Germany (EMBL). The Illumina MiSeq platform was used for sequencing against the reference genome sequence of *N. meningitidis* strain MC58. The result was overlapping sequences approximately 300 bp in length. The genome assembly from primary raw WGS data was performed using the Velvet *de novo* Assembler software [19]. Genomes of the isolates were registered in the PubMLST database (https://pubmlst.org/), using the BIGSdb (Bacterial Isolate Genome Sequence Database) platform [20, 21]. In the PubMLST database, automated genome scanning was then done followed by characterization of gene allelic profile – finetyping loci (*porA*, *fetA*) [22], MLST genes (*abcZ*, *adk*, *aroE*, *fumC*, *gdh*, *pdhC*, and *pgm*) [23], MenB vaccine antigen genes (*nhba*, *nadA*, and *fHbp*) [24] and ribosomal genes [25]. New gene and peptide variants were scanned manually, and after approval and annotation, were added to the PubMLST database. Genomes were compared using the BIGSdb Genome Comparator tool with the "all loci" scheme, which provides the highest resolution. Incomplete loci (due to incomplete assembly) were ignored in pairwise in the distance matrix calculations. The distance matrices, which are based on the number and allelic variability of the genes contained in individual schemes, were generated automatically and phylogenetic networks were constructed using the SplitsTree4 software with uses the NeighborNet algorithm [26]. Phylogenetic analysis results were graphically edited by the Inkscape tool (www.inkscape.org/en/).

Results

Analysis of WGS data on Czech isolates of *N. meningitidis* Y from 1993-2018

WGS assigned 36 of 43 isolates of *N. meningitidis* Y to five clonal complexes: cc23, cc92, cc167, cc103, and cc174, while seven isolates remained unassigned to any of the described clonal complexes (ccUA) (Table 1).

Table 1. Molecular characterization of *N. meningitidis* Y isolates collected in the Czech Republic from 1993 to 2018, (n = 43).

Strain	Year of isolation	PubMLST IDs	Symptoms	Sero group	Geno group	*porA* VR1	*porA* VR2	*fetA* VR	ST	*abcZ*	*adk*	*aroE*	*fumC*	*gdh*	*pdhC*
**0059/93**	1993	**966**	CAR	**Y**	**Y**	5-1	10-14	F5-14	**92**	3	7	4	37	8	18
**0111/93**	1993	**1580**	CAR	**Y**	**Y**	18-7	9	F5-13	**130**	23	5	9	3	13	32
**0224/93**	1993	**1614**	CAR	**Y**	**Y**	5-1	10-4	F1-5	**94**	3	7	4	35	8	18
**0323/93**	1993	**8142**	CAR	**Y**	**Y**	5-1	10-1	F5-14	**92**	3	7	4	37	8	18
**0338/93**	1993	**8152**	CAR	**Y**	**Y**	5-1	10-1	F5-14	**92**	3	7	4	37	8	18
**0200/94**	1994	**83899**	PAC	**Y**	**Y**	5-1	10-4	F1-5	**92**	3	7	4	37	8	18
**0040/95**	1995	**83871**	PAC	**Y**	**Y**	5-1	2-2	F5-8	**569**	10	5	93	14	11	9
**0449/95**	1995	**83903**	PAC	**Y**	**Y**	5-1	10-4	F1-5	**92**	3	7	4	37	8	18
**0008/96**	1996	**83865**	PAC	**Y**	**Y**	12-1	16-8	F5-13	**130**	23	5	9	3	13	32
**0264/99**	1999	**83901**	PAC	**Y**	**Y**	5-1	2-2	F5-8	**1625**	10	5	18	14	11	9
**0172/01**	2001	**83895**	PAC	**Y**	**Y**	5-1	2-2	F5-8	**1625**	10	5	18	14	11	9
**0179/01**	2001	**83897**	PAC	**Y**	**Y**	5-1	2-2	F5-8	**569**	10	5	93	14	11	9
**0251/01**	2001	**83900**	PAC	**Y**	**Y**	5-2	10-1	F4-1	**23**	10	5	18	9	11	9
**0055/02**	2002	**83874**	PAC	**Y**	**Y**	7-2	13-2	F4-17	**23**	10	5	18	9	11	9
**0068/02**	2002	**83877**	PAC	**Y**	**Y**	5-1	2-2	F5-8	**1625**	10	5	18	14	11	9
**0177/02**	2002	**83896**	PAC	**Y**	**Y**	5-1	2-2	F5-8	**569**	10	5	93	14	11	9
**0066/03**	2003	**83911**	PAC	**Y**	**Y**	18-	10-2	F2-	**130**	23	5	9	3	13	32

						12		7							
**0157/03**	2003	**83922**	PAC	**Y**	**Y**	5-2	10-1	F5-8	**3015**	219	5	275	17	11	8
**0192/04**	2004	**83898**	PAC	**Y**	**Y**	5-1	2-2	F5-8	**1625**	10	5	18	14	11	9
**0267/04**	2004	**83902**	PAC	**Y**	**Y**	5-1	2-2	F5-8	**569**	10	5	93	14	11	9
**0065/05**	2005	**83910**	PAC	**Y**	**Y**	5-2	10-1	F5-8	**3015**	219	5	275	17	11	8
**0075/05**	2005	**83913**	PAC	**Y**	**Y**	5-1	2-2	F5-8	**1625**	10	5	18	14	11	9
**0094/07**	2007	**83915**	PAC	**Y**	**Y**	5-2	10-1	F4-1	**23**	10	5	18	9	11	9
**0105/07**	2007	**83917**	PAC	**Y**	**Y**	18-12	10-2	F2-7	**130**	23	5	9	3	13	32
**0156/07**	2007	**83921**	PAC	**Y**	**Y**	18-1	16-4	F3-4	**3015**	219	5	275	17	11	8
**0190/07**	2007	**83923**	PAC	**Y**	**Y**	5-1	10-4	F4-1	**168**	2	16	6	17	9	18
**0121/08**	2008	**83920**	PAC	**Y**	**Y**	5-2	10-1	F4-1	**23**	10	5	18	9	11	9
**0125/08**	2008	**106031**	CAR	**Y**	**Y**	5-2	10-1	F4-1	**23**	10	5	18	9	11	9
**0126/08**	2008	**106032**	CAR	**Y**	**Y**	5-2	10-1	F4-1	**23**	10	5	18	9	11	9
**0049/09**	2009	**83872**	PAC	**Y**	**Y**	5-2	10-1	F4-1	**23**	10	5	18	9	11	9
**0039/10**	2010	**83870**	PAC	**Y**	**Y**	5-1	10-4	F4-1	**168**	2	16	6	17	9	18
**0063/10**	2010	**83876**	PAC	**Y**	**Y**	5-2	10-1	F5-12	**8526**	10	344	18	9	11	9
**0004/11**	2011	**83864**	PAC	**Y**	**Y**	5-2	10-1	F4-1	**23**	10	5	18	9	11	9
**0014/11**	2011	**83904**	CAR	**Y**	**Y**	5-1	2-2	F5-8	**1625**	10	5	18	14	11	9
**0102/11**	2011	**83916**	PAC	**Y**	**Y**	5-1	10-4	F4-1	**168**	2	16	6	17	9	18
**0089/12**	2012	**83880**	PAC	**Y**	**Y**	5-1	10-4	F4-1	**168**	2	16	6	17	9	18
**0032/13**	2013	**83868**	PAC	**Y**	**Y**	5-2	10-1	F4-1	**23**	10	5	18	9	11	9
**0042/13**	2013	**36323**	PAC	**Y**	**Y**	5-2	10-1	F5-12	**23**	10	5	18	9	11	9
**0060/14**	2014	**83875**	PAC	**Y**	**Y**	7-2	13-2	F3-9	**11017**	8	5	6	17	8	18
**0024/16**	2016	**83867**	PAC	**Y**	**Y**	5-1	10-4	F4-1	**168**	2	16	6	17	9	18
**0019/17**	2017	**83866**	PAC	**Y**	**Y**	5-1	10-4	-	**168**	2	16	6	17	9	18
**0037/18**	2018	**101296**	PAC	**Y**	**Y**	5-1	10-4	F1-5	**92**	3	7	4	37	8	18
**0061/18**	2018	**82068**	PAC	**Y**	**Y**	21	16	F3-7	**1466**	6	5	173	13	5	24

VR1, VR2 = *porA* variable region 1 and 2; VR = *fetA* variable region; ST = sequence type; cc = clonal complex, UA = clonal complex unassigned; yellow highlight = newly described allele, sequence type, ribosomal sequence type, or the BAST type; MenDeVAR: green,

orange, grey, and red highlight = graphical representation of four groups with different functional effects in relation to both MenB vaccines

Eighteen isolates from 35 invasive belonged to clonal complex cc23, which was detected throughout the studied years, but showed no increase in any period (S2 Fig). The occurrence of cc23 was recorded in all age groups of IMD patients, being the highest in 15-19-year-olds (S3 Fig).

The phylogenetic network (Fig 1) illustrates the relatedness of all study isolates of *N. meningitidis* Y from the Czech Republic (n = 43).

Fig 1. Genetic relationship of *N. meningitidis* Y isolates collected in the Czech Republic from 1993 to 2018, (n = 43).

Neighbour-net network showing the relatedness among the 43 studied MenY isolates. Isolates are coloured according to detection year and labelled by their NRL number, ST, and cc. Invasive isolates are marked with a square, carriage isolates with a circle.

The genetic relationships between cc23 isolates are shown in more detail in a separate phylogenetic network (Fig 2).

Fig 2. Genetic relationship of *N. meningitidis* Y isolates collected in the Czech Republic from 1993 to 2018, clonal complex cc23, (n = 21).

Neighbour-net network of MenY isolates cc23. Isolates are coloured according to detection year and labelled by their NRL number, ST, and cc. Invasive isolates are marked with a square, carriage isolates with a circle.

The most represented clonal complex in our study set is cc23 (n = 21), which forms a separate lineage, distinct from all other isolates of *N. meningitidis* Y. The cc23 lineage quite rapidly splits into two sister sublineages, ST-23 and ST-569/1625. The study isolates assigned to cc23 belong to four different sequence types: ST-23 (n = 10), ST-569 (n = 4), ST-1625 (n = 6), and ST-8526 (n = 1). Eight of 10 ST-23 isolates (six invasive and two carriage isolates) form a highly compact cluster of sublineage ST-23, within which there are only minimal genetic differences. All eight isolates had the same finetype (P1.5-2,10-1: F4-1) and BAST-228. Except isolate 94/07 (new rST-137531), all isolates of this cluster show ribosome profile rST-2421. Two related invasive isolates, 42/13 (ST-23; rST-89807) and 63/10 (ST-8526; rST-7645), diverge from cluster ST-23, sharing the same finetype (P1.5-2,10-1: F5-12) and BAST-228. Sequence type ST-8526 differs from ST-23 in the MLST genes by allele 344 of the *adk* gene, which was newly described in isolate 63/10 as well as ST-8526 itself. The other sublineage is formed by two genetically related clusters, ST-569 and ST-1625, which include exclusively invasive isolates of the respective sequence types (the only exception is the carrier isolate 14/11, ST-1625). Sequence types ST-569 and ST-1625 contain the *fumC* gene represented by allele 14 in contrast to ST-23 which carries *fumC* allele 9. ST-569 isolates show an additional change in the *aroE* gene (allele 93). All ST-569 and ST-1625 isolates had the same finetype (P1.5-1,2-2: F5-8) and BAST-227. Except three isolates with newly defined ribosomal sequence types: 264/99 (rST-137525), 179/01 (rST-137523), and 68/02 (rST-137521) and isolate 267/04 (rST-2589), isolates ST-569 and ST-1625 share ribosome profile rST-2422. Interestingly, invasive isolate 55/02 - Y: P1.7-2,13-2: F4-17: ST-23 (cc23), despite its standard MLST gene alleles, is the only cc23 outlier from sublineages ST-23 and ST-569/1625. A more detailed representation shows that it has a unique genetic profile and is unrelated to any of the major sublineages of clonal complex cc23. In this isolate, a new

ribosomal sequence type, rST-137520, and a combination of MenB vaccine antigen genes, BAST-2988, have also been described.

The second leading clonal complex of the study isolates of *N. meningitidis* Y is cc92. It is represented by three invasive and four carriage isolates and is quite heterogeneous in the phylogenetic network. One lineage of clonal complex cc92 is formed by two almost identical carriage isolates, 323/93 and 338/93 (P1.5-1,10-1: F5-14: ST-92 (cc92); rST-3006; BAST-987), along with a more distant carriage isolate 59/93 (ST-92). This isolate exhibits differences in the finetype (P1.5-1,10-14: F5-14) and ribosome profile (rST-3142) and is of BAST type which was described for the first time (BAST-2917). Another lineage of cc92, groups two genetically closely related invasive isolates, 200/94 and 449/95 (P1.5-1,10-4: F1-5: ST-92 (cc92); new rST-137524; BAST-1501). A more distant relatedness to this lineage was evidenced for a recent invasive isolate, 37/18 (ST-92), which was assigned to the same finetype and BAST-1501. The genetic difference of this isolate is due to ribosomal gene alleles, and their specific combination was registered in the PubMLST database as a new ribosomal sequence type, rST-163667. The list of isolates of clonal complex cc92 ends with an outlier carriage isolate, 224/93 (P1.5-1,10-4: F1-5; rST-3214; BAST-549), which is the only one to be assigned to a different sequence type, ST-94. This combination of MLST alleles differs from ST-92 in allele variants of the *fumC* (35) and *pgm* (2) genes. Except isolate 37/18, recovered in 2018, all cc92 isolates were only detected in the Czech Republic between 1993 and 1995.

Six highly related invasive isolates form lineage cc167 in the phylogenetic network, which shows relatedness to lineage cc92. All cc167 isolates carry a single sequence type, ST-168, and BAST-384. Except isolate 24/16 with a newly described ribosomal profile, rST-125578,

all cc167 isolates share rST-7517. All but one isolates are of the same finetype (P1.5-1,10-4: F4-1). In isolate 19/17, a single nucleotide deletion occurred in the variable region (VR) of the *fetA* gene, resulting in reading frame shift and internal stop codon emergence. This mutated *fetA* allele, originating from active allele 607 and unable to produce functional peptide, was registered in the PubMLST as allele 2816. Four of six study cc167 isolates were collected after 2010, and from the phylogenetic network, it is evident that cc167 has recently become more common among the isolates of *N. meningitidis* Y.

Another comparatively heterogeneous lineage groups four isolates ST-130 unassigned to clonal complex (ccUA). Two invasive isolates showing two newly described ribosome profiles, 66/03 (rST-137529) and 105/07 (rST-137532), share the same finetype (P1.18-12,10-2: F2-7) and BAST-1298. For isolate 105/07, a new allele variant of the *fHbp* gene (1079) was described, which differs from the original allele 302 by two synonymous substitutions, thus encoding the same peptide variant, 245. The third invasive isolate 8/96 (P1.12-1,16-8: F5-13: ST-130 (ccUA); new rST-137518), for which BAST-2987 was newly defined, shows a slightly higher relatedness to the carriage isolate from 1993, 111/93 (P1.18-7,9: F5-13: ST-130 (ccUA); rST-3297; BAST-1479) as compared to other invasive isolates ST-130.

An outlier invasive isolate of clonal complex cc103 - 60/14 (P1.7-2,13-2: F3-9) appears to be distantly related to the lineage of isolates ST-130. Based on this outlier, a new sequence type, ST-11017 (a new combination of known MLST alleles), a new ribosomal sequence type, rST-169886, and a new combination of MenB vaccine antigen genes, BAST-2972, were defined.

Even more distant relatedness to isolates ST-130 is seen in a lineage of three isolates ST-3015 (ccUA) recovered between 2003 and 2007. This lineage consists of two highly related

isolates, 157/03 and 65/05 (P1.5-2,10-1: F5-8; BAST-1418), which molecularly only differ in ribosome profiles. While isolate 157/03 belongs to rST-7234, isolate 65/05 is of rST-137528, newly described in it. Molecular characteristics of isolate 156/07 differ substantially from those of the other two isolates ST-3015 (P1.18-1,16-4: F3-4; ST-3015 (ccUA); new rST-137534), which is consistent with its position in the phylogenetic network. Based on isolate 156/07, BAST-2990 was newly defined.

The last isolate from our 2018 collection – 61/18 (P1.21,16: F3-7; ST-1466 (cc174); rST-80732; BAST-14) is a phylogenetic outlier and does not show any relatedness to other isolates of *N. meningitidis* Y.

In addition to BAST (Bexsero antigen sequence type), which is defined in the PubMLST database as a combination of allele variants of two variable regions of the *porA* genes (VR1 and VR2) and peptide variants of three MenB-4C vaccine antigen genes (*nhba*, *nadA*, and *fHbp*), thus providing information on theoretical coverage of a given isolate by the Bexsero vaccine, the Meningococcal Deduced Vaccine Antigen Reactivity (MenDeVAR) Index providing information on the functional effect of both MenB vaccines (Bexsero and Trumenba) for a given isolate has recently been introduced in the PubMLST database. The MenDeVAR index is based on the analysis of data on the presence of particular allele variants of MenB vaccine antigens, their expression, and bactericidal susceptibility test result [27]. According to the MenDeVAR index, the isolates are classified into four groups in relation to both MenB vaccines. Isolates containing one or more specific antigenic variants included in MenB vaccines are highlighted in green. Isolates containing one or more antigenic variants that showed cross-reactivity in experimental studies are highlighted in orange. Isolates for which enough data have not been available on their antigenic variants are highlighted in grey,

and those carrying antigenic variants that did not show cross-reactivity in experimental studies are highlighted in red.

Given the type of construction of the peptide MenB vaccines, among other benefits, they have the potential to provide protection against other meningococcal serogroups. In the study collection of MenY isolates, this phenomenon can be observed particularly for Trumenba (Table 1). The MenDeVAR index classified 32 of 43 isolates (nearly 75 %) in the PubMLST database as covered by cross-reactivity. Experimental data have not yet been available for the remaining 11 isolates. Regarding the Bexsero vaccine one isolate carried *nadA* allele variant 8, which is included in this vaccine. One isolate appeared to be covered due to cross-reactivity and one isolate was considered as uncovered by the Bexsero vaccine based on the MenDeVAR index. For the remaining 40 isolates, experimental data for at least one antigenic variant were not available, and thus they could not be categorized based on the MenDeVAR index.

Analysis of WGS data on Czech and European isolates of *N. meningitidis* Y

The phylogenetic network (Fig 3A and Fig 3B) shows the levels of relatedness between all European invasive isolates of *N. meningitidis* Y, including Czech isolates, which were available in the PubMLST database by May 26, 2021 and met the following criteria: continent Europe, serogroup Y, genogroup Y, invasiveness, complete MLST profile, and sequencing data size of > 1.8 MB. These were 995 isolates analysed by WGS from the countries below: UK (n = 612), Sweden (n = 112), France (n = 62), Germany (n = 49), the Netherlands (n = 46), Czech Republic (n = 35), Ireland (n = 29), Austria (n = 12), Norway (n = 11), Italy (n = 10), Finland (n = 6), Slovenia (n = 6), Malta (n = 3), Cyprus (n = 1), and Greece (n = 1).

Fig 3A. Genetic diversity of *N. meningitidis* Y invasive isolates from the Czech Republic and European countries, described by years of isolation (n = 995).

Isolates are coloured according to detection year. Invasive isolates from Czech Republic (n = 35) are marked with a dark blue square and labelled by their NRL number, ST, and cc.

Fig 3B. Genetic diversity *of N. meningitidis* Y invasive isolates from the Czech Republic and European countries, described by country of origin (n = 995).

Isolates are coloured according to the country of origin. Invasive isolates from Czech Republic (n = 35) are marked with a dark blue square and labelled by their NRL number, ST, and cc.

The general view shows that one part of the European isolates of *N. meningitidis* Y form a large subpopulation of several lineages of clonal complex cc23 in the phylogenetic network, while the other part form separate genetic lineages on the opposite side of the network consistent with the respective clonal complexes.

The large cc23 subpopulation consisting of several more or less related lineages includes 18 invasive isolates of clonal complex cc23 from the Czech Republic. All cc23 lineages are quite evenly represented by isolates from all time periods, and none of them can be considered as historical or modern, but an identical phylogenetic network that shows geographical origin of isolates in colour provides a completely different view. An outlier lineage can be noticed (I.), grouping almost exclusively ST-23 isolates from the UK. In three similar and genetically closely related lineages (II., III., and IV.), the UK isolates are dominant again; however,

isolates from neighbouring countries are also significantly represented (in particular those from Scotland, France, the Netherlands, and Germany). Most isolates from these three lineages belong to sequence type ST-1655. Another cc23 lineage comprises mostly Swedish ST-23 isolates (V.). Close to the Swedish lineage is located a geographically diverse lineage (VI.) of isolates from continental Europe where ST-23 and sequence types derived from it are dominant. Apart from a cluster of six Czech isolates (251/01, 94/07, 121/08, 49/09, 4/11, and 32/13), this lineage includes primarily isolates from Germany, Austria, the Netherlands, and Slovenia. Czech isolates 63/10 and 42/13, which do not cluster together, belong to a genetically more distant lineage comprising mostly ST-23 isolates from France (VII.). The list of cc23 lineages ends with two outlier lineages, one smaller and comparatively homogeneous, grouping isolates from Italy, France, UK, and the Netherlands (VIII.), and the other larger and more heterogeneous (IX.). It is composed mainly of German, British, Norwegian, Swedish, and Czech isolates and splits into a number of separate sublineages. One of these sublineages is formed by two genetically closely related clusters (ST-569 and ST-1625) of 9 invasive isolates from the Czech Republic along with UK isolate M16_240445 from 2016 (ST-1625). ST-23 is also dominant in these two lineages. The last Czech cc23 isolate is 55/02 (ST-23), which, similar to the previous Czech phylogenetic network, is an outlier, and from its position, it is evident that it is highly genetically distant from all other European cc23 isolates.

Nor do European isolates of *N. meningitidis* Y not belonging to cc23 show any significant trend in time distribution. The phylogenetic network which defines these isolates as genetically highly distant from cc23 subpopulation rapidly splits into three major lineages. The first one is compact lineage cc174, consisting exclusively with the exception of two isolates (ST-7867 a ST-9893) of ST-1466 isolates. Lineage cc174 includes two sister sublineages, one groups mostly isolates from UK and Scotland and the other comprises

isolates from continental Europe (Germany, France, and the Netherlands), including isolate 61/18 (ST-1466) from the Czech Republic. The latter lineage is highly heterogeneous, both geographically and genetically. It groups isolates ccUA, cc22, cc103, cc11, and cc865. The highest number of isolates within this lineage split into two sublineages. Sublineage cc22, consisting of UK isolates and a cluster of three isolates from Germany, is adjacent to the geographically diverse sublineage ccUA. Sublineage ccUA also includes three isolates from the Czech Republic: 157/03, 65/05, and 156/07 (ST-3015). Five separate sublineages represented by only a few isolates diverge from the second major lineage just at its beginning. One of these sublineages comprises a cluster of three Czech isolates ST-130. Another sublineage groups two isolates ST-9316, ccUA (Germany, France), UK isolate ST-3342, cc865, and Czech isolate 60/14 (ST-11017, cc103). Interestingly, sublineage cc11 groups together one Swedish and two UK isolates ST-11 and outlier UK isolate M14_240327 (ST-910, ccUA). The third major lineage in terms of heterogeneity appears in between the two former lineages. At the bottom, a sublineage of five cc92 isolates separates from it, formed by a cluster of three Czech isolates ST-92 (200/94, 449/95, and 37/18) along with a UK isolate, M11_240437 (ST-784), and a Dutch isolate, IBD-830, which belongs to the same sequence type. Other members of this lineage are almost exclusively isolates cc167. In the cc167 lineage, several sublineages can be noticed again, consisting of isolates according to sequence type and geographical origin: sublineage ST-767 mostly represented by German isolates, UK-French sublineage ST-1627, and sublineage ST-1624 mostly consisting of isolates from UK and Scotland. The last sublineage, ST-168, groups mainly UK isolates along with a cluster of six ST-168 isolates from the Czech Republic.

In conclusion, the European invasive isolates of *N. meningitidis* Y, including Czech isolates, can be considered as a considerably heterogeneous population, about two thirds of which are

genetically closely related and belong to cc23. The remaining isolates are assigned to a number of other clonal complexes and show a very low relatedness to cc23 isolates and to each other. Isolates of *N. meningitidis* Y belonging to cc23 and other clonal complexes form sublineages within genetic lineages, defined mainly geographically. In terms of time, the population of invasive isolates of *N. meningitidis* Y appears to be surprisingly constant. The Czech isolates of *N. meningitidis* Y generally follow the trend observed for European isolates. From the perspective of clonal complexes, they are highly heterogeneous and mostly cluster together within particular genetic lineages.

Analysis of WGS data on Czech and non-European isolates of *N. meningitidis* Y

The last phylogenetic network (Fig 4A and Fig 4B) compares invasive isolates of *N. meningitidis* Y from the Czech Republic and non-European countries. In the PubMLST database, 375 non-European isolates were available by May 26, 2021, meeting the following criteria: non-European country, serogroup Y, genogroup Y, invasiveness, complete MLST profile, and sequencing data size of > 1.8 MB. In this selection, the MenY isolates from the USA are dominant (n = 315), followed by Japan (n = 24), New Zealand (n = 13), Mexico (n = 8), Canada (n = 4), Turkey (n = 3), China (n = 2), Morocco (n = 2), Burkina Faso (n = 2), Chile (n = 1), and Bangladesh (n = 1). Along with 35 isolates from the Czech Republic, the study set comprises 410 invasive isolates.

Fig 4A. Genetic diversity of *N. meningitidis* Y invasive isolates from the Czech Republic and non-European countries, described by years of isolation (n = 410).

Isolates are coloured according to detection year. Invasive isolates from Czech Republic (n = 35) are marked with a dark blue square and labelled by their NRL number, ST, and cc.

Fig 4B. Genetic diversity of *N. meningitidis* Y invasive isolates from the Czech Republic and non-European countries, described by country of origin (n = 410).

Isolates are coloured according to the country of origin. Invasive isolates from Czech Republic (n = 35) are marked with a dark blue square and labelled by their NRL number, ST, and cc.

A general view of the phylogenetic network shows again a large subpopulation of cc23 isolates while isolates of *N. meningitidis* Y of other clonal complexes form several separate ~~more or less~~ numerous lineages on the opposite side.

Unlike the European cc23 isolates, non-European cc23 isolates appear less homogeneous. They form four separate, genetically distinct lineages. Furthermore, they differ in time distribution of isolates in individual lineages. The first cc23 lineage (I~~.~~) comprising exclusively isolates from the USA and Mexico is represented mostly by isolates ST-3587 recovered after 2016. At the base of this lineage, an outlier isolate, M25960, from 2013 can be seen, which still belongs to ST-23. Another cc23 lineage (II~~.~~) groups two main sister sublineages of isolates from the USA, with ST-23 and related ST-3532 being dominant. In addition to the two main sublineages, three other sublineages separate from the lineage II~~.~~ of cc23 isolates. One is formed by a single isolate from the USA (ST-12944) and two isolates from Burkina Faso (ST-4375), the second comprises two Japanese isolates (ST-23), and the third sublineage groups two clusters of 9 isolates from the Czech Republic (ST-569 and ST-1625) along with three isolates from the USA (2x ST-1622 and 1x ST-1625). Unlike the first

major cc23 lineage, the second one is evenly represented by isolates from all time periods. The third cc23 lineage is geographically more diverse (III.). Apart from the dominant isolates from the USA, it includes isolates from Japan, New Zealand, Canada, China, and the Czech Republic, mostly assigned to sequence type ST-23. Two sublineages diverge from this lineage: one with dominant ST-23 groups isolates from the USA along with two Czech isolates (63/10 and 42/13) and sublineage ST-1655 comprising isolates from the USA, Japan, New Zealand, and China. The remaining six Czech ST-23 isolates cluster together within the major lineage. In the third cc23 lineage, isolates show a quite even time distribution, except for sublineage ST-1655 consisting mostly of modern isolates. The last, fourth cc23 lineage (IV.) only includes two isolates, 55/02 (ST-23) from the Czech Republic collected in 2002, which so far occupied a completely separate position in the phylogenetic networks, along with a related American isolate, M28856 (ST-6519) from 2014.

Non-European isolates of *N. meningitidis* Y which do not belong to clonal complex cc23 form again several lineages genetically distant from each other on the opposite side of the phylogenetic network. In comparison with the European isolates, non-European isolates are less represented by other clonal complexes, as e.g., two genetic lineages are only formed by isolates from the Czech Republic. The only exception is richly represented lineage cc167, consisting mainly of American isolates ST-1624. Part of this lineage is a cluster of four isolates ST-1624 from New Zealand, two Canadian isolates (ST-3923 and ST-3980), and one isolate each from Mexico (ST-11176) and Japan (ST-14846). Two sublineages diverge from lineage cc167 – one includes five isolates from the USA (3x ST-2880, 2x ST-767) and one isolate each from Turkey and Bangladesh (both ST-767) and the other is formed by a cluster of six Czech isolates ST-168 along with a genetically rather distant isolate from Morocco (ST-1627). At the base of the cc167 lineage, as was the case with the previous phylogenetic

networks, lineage cc92 separates formed by a cluster of three Czech isolates ST-92. Another lineage is represented exclusively by isolates ST-1466 (cc174), namely isolate 61/18 from the Czech Republic, 12 isolates from the USA, and one isolate from Morocco. The last lineage splits at the base into three separate sublineages. The first one groups seven isolates ST-11 (cc11) from the USA (5x), Turkey (1x), and Chile (1x). In the second sublineage, dominant are the isolates of sequence type ST-3015 (ccUA) from the Czech Republic (3x), USA (1x), and Japan (1x). Three more isolates are part of this group: one each from Japan (ST-5168, ccUA), New Zealand (ST-5436, ccUA), and Turkey (ST-12009, cc22). The last sublineage comprises exclusively Czech isolates, a cluster of three isolates ST-130 (ccUA) and isolate 60/14 (ST-11017, cc103), which is quite distantly related to isolates ST-130.

The non-European population of isolates of *N. meningitidis* Y appears to be less diverse in terms of clonal complexes as compared to the European one. Most isolates belong to complexes cc23 and cc167. A higher genetic diversity can be seen within individual clonal complexes – particularly in subpopulation cc23. The non-European population of *N. meningitidis* Y also shows a different time distribution of isolates. Within clonal complexes, several modern sublineages appear, containing mostly isolates collected after 2016. The Czech isolates of *N. meningitidis* Y, added to the non-European isolates for comparison, do not follow the observed non-European trend. Their high clonal heterogeneity considerably contributes to the overall heterogeneity of the phylogenetic network, which contains Czech and non-European isolates.

Discussion

The analysis of WGS data on Swedish IMD isolates of *N. meningitidis* Y from 1995-2012 revealed two major branches of clonal complex cc23, one of which corresponded to YI strain type and the other to YII and YIII strain types [14]. The comparison of the Swedish WGS data with those from the England, Wales, and Northern Ireland confirmed the spread of these two branches across continents over the last few years, although these branches were referred to differently in studies. Detailed analysis of WGS data shows differences between variants of each cluster in one or a few loci, most of which occurred only in Swedish isolates. The recent rise in IMD caused by serogroup Y in Sweden was due primarily to YI strain type, belonging to WGS lineage 23.1. These isolates were antigenically indistinguishable, but the cgMLST analysis demonstrated two different clusters (subtype 1 and subtype 2). These results suggest that the rise in IMD can be caused by a new subtype of cc23 and its rapid spread in the country.

IMD isolates of serogroup Y collected in England and Wales in 2007-2009 belonged to four major clonal complexes: cc23 (56 %), cc174 (21 %), cc167 (11 %), and cc22 (8 %). The rise in IMD caused by serogroup Y in 2009 was mainly due to ST-1655 (cc23) [10].

The variability of *N. meningitidis* Y cc23 was also reported for Italian IMD isolates from 2007-2013, assigned to nine different sequence types ST (ST-23, ST-10098, ST-1665, ST-10348, ST-9326, ST-2533, ST-9253, ST-3171, and ST-2692), with a predominance of ST-23 [13]. In our study, ST-23 was also prevalent among isolates of *N. meningitidis* Y cc23, but other STs (ST-1625, ST-569, and ST-8526) differed from those identified in the Italian study.

N. meningitidis Y cc23 was responsible for the rise in IMD in the United States during the 1990s. Whole genome pyrosequencing made it possible to analyse the genetic differences

between the early and late populations of *N. meningitidis* Y cc23. Isolates from both periods had similar genomes, but differences were found in the proteins that influence the pilus structure and in proteins involved in iron acquisition. These changes mirror the acquisition of new alleles as a result of horizontal gene transfer [9].

IMD isolates of serogroup Y collected in Canada in 1999-2003 were assigned to two major clonal complexes: cc23 and cc167. The cc23 isolates showed a high prevalence of ST-23, followed by ST-1625, while other STs tend to be much less common (ST-3704, ST-183, ST-5730, ST-5733, ST-569, and ST-3981). The most frequent ST in the cc167 isolates was ST-3923, and other STs were identified less often (ST-167, ST-3980, ST-1624, ST-572, ST-3949, ST-168, ST-2415, and ST-3705) [28]. The prevalence of clonal complexes cc23 and cc167 found in our study is consistent with the Canadian data, as is the prevalence of ST-23 in clonal complex cc23. Our results only differ from theirs in the number of sequence types in clonal complex cc167: in the Czech group, only ST-168 was detected compared to nine different STs in the Canadian cc167 isolates.

The comparison of IMD isolates of serogroup Y from the United States, South Africa, and Israel collected in 1999-2002 showed the prevalence of two clonal complexes, cc23 and cc175. However, differences were found in the representation of clonal complexes between countries: the USA and Israel reported the prevalence of cc23 (94 % and 96 %, respectively), while in South Africa, the prevalence of cc175 (77 %) was observed [29].

Molecular characterization of IMD isolates of *N. meningitidis* Y collected in Latin American countries in 2000-2006 indicated the prevalence of clonal complexes cc23 and cc167, varying between countries: cc23 was prevalent in Columbia and Costa Rica while the prevalence of

cc167 was reported from Argentina [8]. In our study, cc23 was the leading clonal complex and cc167 was the second leading complex among the isolates of *N. meningitidis* Y.

WGS analysis of isolates of *N. meningitidis* Y from healthy carriers collected in the United Kingdom in 1997-2010 and IMD isolates from 2010-2011 assigned 91 % of these isolates to one of eight clusters. Six clusters grouped carriage isolates from 1997-2001, which suggests long-term temporal stability and an overlap of the carriage and IMD populations for the clones of *N. meningitidis* Y. However, one of the eight clusters, generally with the characteristics Y: P1.5-1,10-1: F4-1; ST-1655 (cc23), splits into two subclusters, one of which groups 86 % of carriage isolates and the other 90 % of IMD isolates, thus suggesting the existence of disease-associated clones [30].

In conclusion, the most represented clonal complex in Czech *N. meningitidis* Y isolates is cc23, which forms a separate lineage, distinct from all other isolates of *N. meningitidis* Y. The WGS analysis showed the population of Czech *N. meningitidis* Y isolates as very heterogeneous, containing a large number of unrelated genetic lineages. The Czech isolates of *N. meningitidis* Y generally follow the trend observed for European isolates and do not follow the observed non-European trend.

Acknowledgements

This publication made use of the PubMLST website (https://pubmlst.org/) developed by Keith Jolley (Jolley & Maiden 2010, BMC Bioinformatics, 11:595) and sited at the University of Oxford. The development of that website was funded by the Wellcome Trust.

References

1. Goldacre MJ, Roberts SE, Yeates D. Case fatality rates for meningococcal disease in an English population, 1963-98: database study. BMJ. 2003 Sep 13; 327(7415):596-7. DOI: 10.1136/bmj.327.7415.596. PMID: 12969927.
2. Sharip A, Sorvillo F, Redelings MD, Mascola L, Wise M, Nguyen DM. Population-based analysis of meningococcal disease mortality in the United States: 1990-2002. Pediatr Infect Dis J. 2006 Mar; 25(3):191-4. DOI: 10.1097/01.inf.0000202065.03366.0c. PMID: 16511378.
3. Parikh SR, Campbell H, Bettinger JA, Harrison LH, Marshall HS, Martinon-Torres F, *et al*. The everchanging epidemiology of meningococcal disease worldwide and the potential for prevention through vaccination. J Infect. 2020 Oct; 81(4):483-98. DOI: 10.1016/j.jinf.2020.05.079. PMID: 32504737.
4. Stephens DS, Greenwood B, Brandtzaeg P. Epidemic meningitis, meningococcaemia, and *Neisseria meningitidis*. Lancet. 2007 Jun 30; 369(9580):2196-210. DOI: 10.1016/S0140-6736(07)61016-2. PMID: 17604802.
5. Whitaker R, Gomes Dias J, Ramliden M, Ködmön C, Economopoulou A, Beer N, *et al*. The epidemiology of invasive meningococcal disease in EU/EEA countries, 2004–2014. Vaccine. 2017 Apr 11; 35(16):2034-41. DOI: 10.1016/j.vaccine.2017.03.007. PMID: 28314560.
6. European Centre for Disease Prevention and Control. Invasive meningococcal disease. In: ECDC. Annual epidemiological report for 2017. Stockholm: ECDC; 2019.
7. Tsang RSW, Squires SG, Zollinger WD, Ashton FE. Distribution of serogroups of *Neisseria meningitidis* and antigenic characterization of serogroup Y meningococci in

Canada, January 1, 1999 to June 30, 2001. The Canadian Journal of Infectious Diseases. 2002 Nov; 13(6):391-6. DOI: 10.1155/2002/891673. PMID: 18159416.

8. Abad R, Agudelo CI, Brandileone C, Chanto G, Gabastou JM, Hormazabal JC, *et al*. Molecular characterization of invasive serogroup Y *Neisseria meningitidis* strains isolated in the Latin America region. J Infect. 2009 Aug; 59(2):104-14. DOI: 10.1016/j.jinf.2009.06.001. PMID: 19576638.

9. Krauland MG, Dunning Hotopp JC, Riley DR, Daugherty SC, Marsh JW, Messonnier NE, *et al*. Whole genome sequencing to investigate the emergence of clonal complex 23 *Neisseria meningitidis* serogroup Y disease in the United States. PLoS One. 2012; 7(4):e35699. DOI: 10.1371/journal.pone.0035699. PMID: 22558202.

10. Ladhani SN, Lucidarme J, Newbold LS, Gray SJ, Carr AD, Findlow J, *et al*. Invasive Meningococcal Capsular Group Y Disease, England and Wales, 2007–2009. Emerg Infect Dis. 2012 Jan; 18(1):63-70. DOI: 10.3201/eid1801.110901. PMID: 22261040.

11. Törös B, Hedberg ST, Jacobsson S, Fredlund H, Olcén P, Mölling P. Surveillance of invasive *Neisseria meningitidis* with a serogroup Y update, Sweden 2010 to 2012. Euro Surveill. 2014 Oct 23; 19(42):20940. DOI: 10.2807/1560-7917.es2014.19.42.20940. PMID: 25358044.

12. Bröker M, Emonet S, Fazio C, Jacobsson S, Koliou M, Kuusi M, *et al*. Meningococcal serogroup Y disease in Europe: Continuation of high importance in some European regions in 2013. Hum Vaccin Immunother. 2015; 11(9):2281-6. DOI: 10.1080/21645515.2015.1051276. PMID: 26036710.

13. Fazio C, Neri A, Renna G, Vacca P, Antonetti R, Barbui AM, *et al*. Persistent occurrence of serogroup Y/sequence type (ST)-23 complex invasive meningococcal disease among patients aged five to 14 years, Italy, 2007 to 2013. Euro Surveill. 2015; 20(45):30061. DOI: 10.2807/1560-7917.ES.2015.20.45.30061. PMID: 26606870.

14. Törös B, Hadberg ST, Unemo M, Jacobsson S, Hill DMC, Olcén P, *et al*. Genome-Based Characterization of Emergent Invasive *Neisseria meningitidis* Serogroup Y Isolates in Sweden from 1995 to 2012. J Clin Microbiol. 2015 Jul; 53(7):2154-62. DOI: 10.1128/JCM.03524-14. PMID: 25926489.
15. Jandova Z, Musilek M, Vackova Z, Kozakova J, Krizova P. Serogroup and Clonal Characterization of Czech Invasive *Neisseria meningitidis* Strains Isolated from 1971 to 2015. PLoS One. 2016 Dec 9; 11(12):e0167762. DOI: 10.1371/journal.pone.0167762. PMID: 27936105.
16. Křížová P, Honskus M, Okonji Z, Musílek M, Kozáková J. Surveillance of invasive meningococcal disease based on whole genome sequencing (WGS), Czech Republic, 2015. Epidemiol Mikrobiol Imunol. 2018; 67(2):64-73. PMID: 30126283. (in Czech).
17. Honskus M, Okonji Z, Musilek M, Kozakova J, Krizova P. Whole genome sequencing of *Neisseria meningitidis* W isolates from the Czech Republic recovered in 1984–2017. PLoS One. 2018 Sep 13; 13(9):e0199652. DOI: 10.1371/journal.pone.0199652. PMID: 30212468.
18. Krizova P, Honskus M. Genomic surveillance of invasive meningococcal disease in the Czech Republic, 2015-2017. PLoS One. 2019 Jul 11; 14(7):e0219477. DOI: 10.1371/journal.pone.0219477. PMID: 31295279.
19. Zerbino DR. Using the Velvet de novo assembler for short-read sequencing technologies. Curr Protoc Bioinformatics. 2010 Sep; 11(5):1-12. DOI: 10.1002/0471250953.bi1105s31. PMID: 20836074.
20. Jolley KA, Chan MS, Maiden MC. MlstdbNet-distributed multi-locus sequence typing (MLST) databases. BMC Bioinformatics. 2004 Jul 1; 5:86. DOI: 10.1186/1471-2105-5-86. PMID: 15230973.

21. Jolley KA, Maiden MC. BIGSdb: Scalable analysis of bacterial genome variation at the population level. BMC Bioinformatics. 2010 Dec 10; 11:595. DOI: 10.1186/1471-2105-11-595. PMID: 21143983.

22. Jolley KA, Brehony C, Maiden MC. Molecular typing of meningococci: recommendations for target choice and nomenclature. FEMS Microbiol Rev. 2007 Jan; 31(1):89-96. DOI: 10.1111/j.1574-6976.2006.00057.x. PMID: 17168996.

23. Maiden MC, Bygraves JA, Feil E, Morelli G, Russell JE, Urwin R, *et al*. Multilocus sequence typing: a portable approach to the identification of clones within populations of pathogenic microorganisms. Proc Natl Acad Sci U S A. 1998 Mar 17; 95(6):3140-5. DOI: 10.1073/pnas.95.6.3140. PMID: 9501229.

24. Vernikos G, Medini D. Bexsero® chronicle. Pathog Glob Health. 2014 Oct; 108(7):305-16. DOI: 10.1179/2047773214Y.0000000162. PMID: 25417906.

25. Jolley KA, Bliss CM, Bennett JS, Bratcher HB, Brehony C, Colles FM, *et al*. Ribosomal multilocus sequence typing: universal characterization of bacteria from domain to strain. Microbiology (Reading), 2012 Apr; 158(4):1005-15. DOI: 10.1099/mic.0.055459-0. PMID: 22282518.

26. Huson DH. SplitsTree: analyzing and visualizing evolutionary data. Bioinformatics. 1998; 14(1):68-73. DOI: 10.1093/bioinformatics/14.1.68. PMID: 9520503.

27. Rodrigues ChMC, Jolley KA, Smith A, Claire Cameron J, Feavers IM, Maiden MCJ. Meningococcal Deduced Vaccine Antigen Reactivity (MenDeVAR) Index: a Rapid and Accessible Tool That Exploits Genomic Data in Public Health and Clinical Microbiology Applications. J Clin Microbiol. 2020 Dec 17; 59(1):e02161-20. DOI: 10.1128/JCM.02161-20. PMID: 33055180.

28. Tsang RSW, Henderson AM, Cameron ML, Tyler SD, Tyson S, Law DKS, *et al*. Genetic and antigenic analysis of invasive serogroup Y *Neisseria meningitidis* isolates

collected from 1999 to 2003 in Canada. J Clin Microbiol. 2007 Jun; 45(6):1753-8. DOI: 10.1128/JCM.02134-06. PMID: 17442798.

29. Whitney AM, Coulson GB, von Gottberg A, Block C, Keller N, Mayer LW, *et al*. Genotypic comparison of invasive *Neisseria meningitidis* serogroup Y isolates from the United States, South Africa, and Israel, isolated from 1999 through 2002. J Clin Microbiol. 2009 Sep; 47(9):2787-93. DOI: 10.1128/JCM.00091-09. PMID: 19571028.
30. Oldfield NJ, Harrison OB, Bayliss ChD, Maiden MCJ, Ala'Aldeen DAA, Turner DPJ. Genomic Analysis of Serogroup Y *Neisseria meningitidis* Isolates Reveals Extensive Similarities Between Carriage-Associated and Disease-Associated Organisms. J Infect Dis. 2016 Jun 1; 213(11):1777-85. DOI: 10.1093/infdis/jiw008. PMID: 26747709.

Supporting information

S1 Fig. Serogroups of *N. meningitidis* causing invasive meningococcal disease, Czech Republic, 1993 - 2018, surveillance data of NRL for meningococcal infections.

S2 Fig. Clonal complexes of *N. meningitidis* Y causing invasive meningococcal disease, Czech Republic, 1993 - 2018, surveillance data.

S3 Fig. Clonal complexes of *N. meningitidis* Y causing invasive meningococcal disease, Czech Republic, 1993 - 2018, age groups, surveillance data.

Click here to access/download;Figure;Fig 1.tif

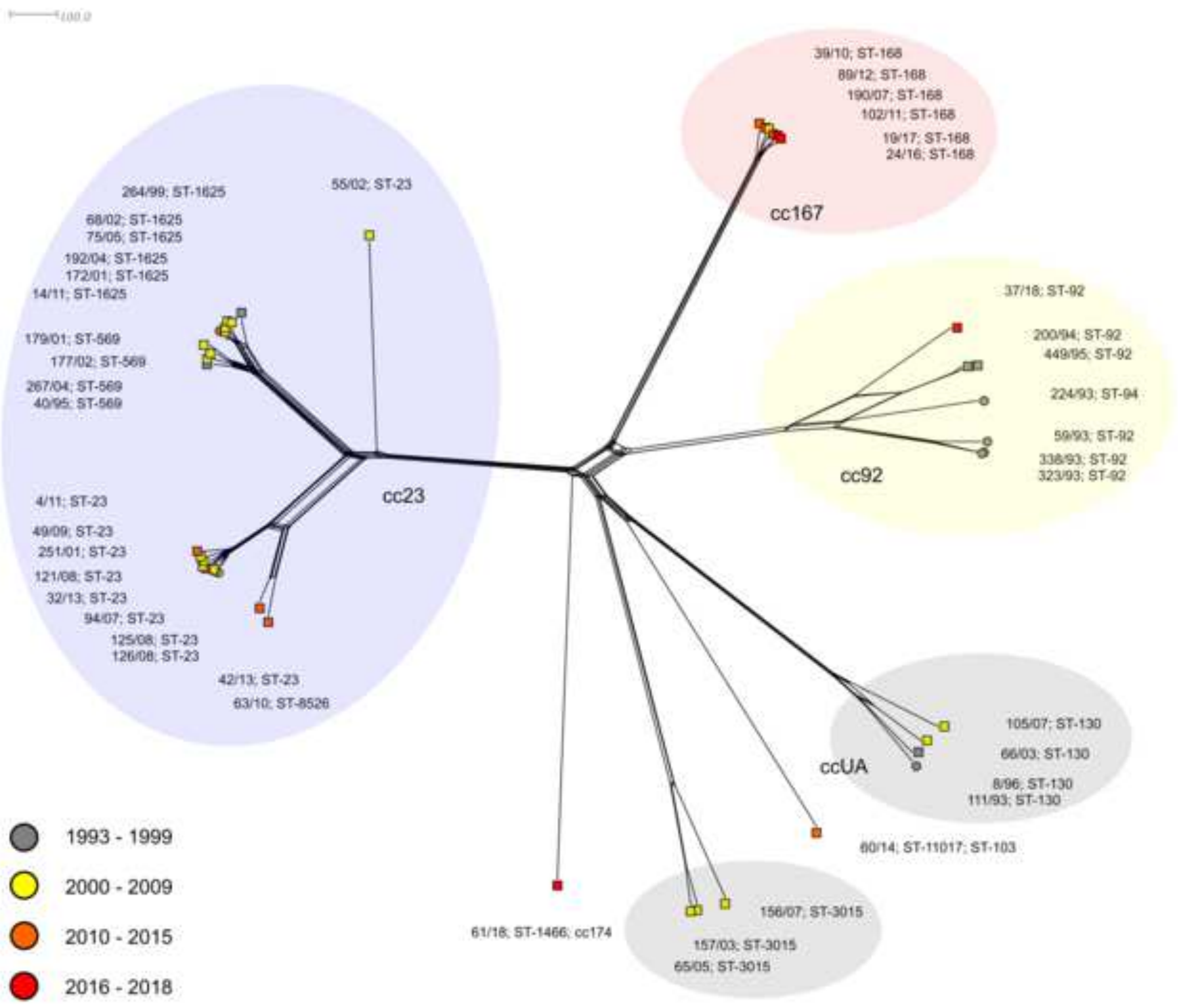

Click here to access/download;Figure;Fig 2.tif

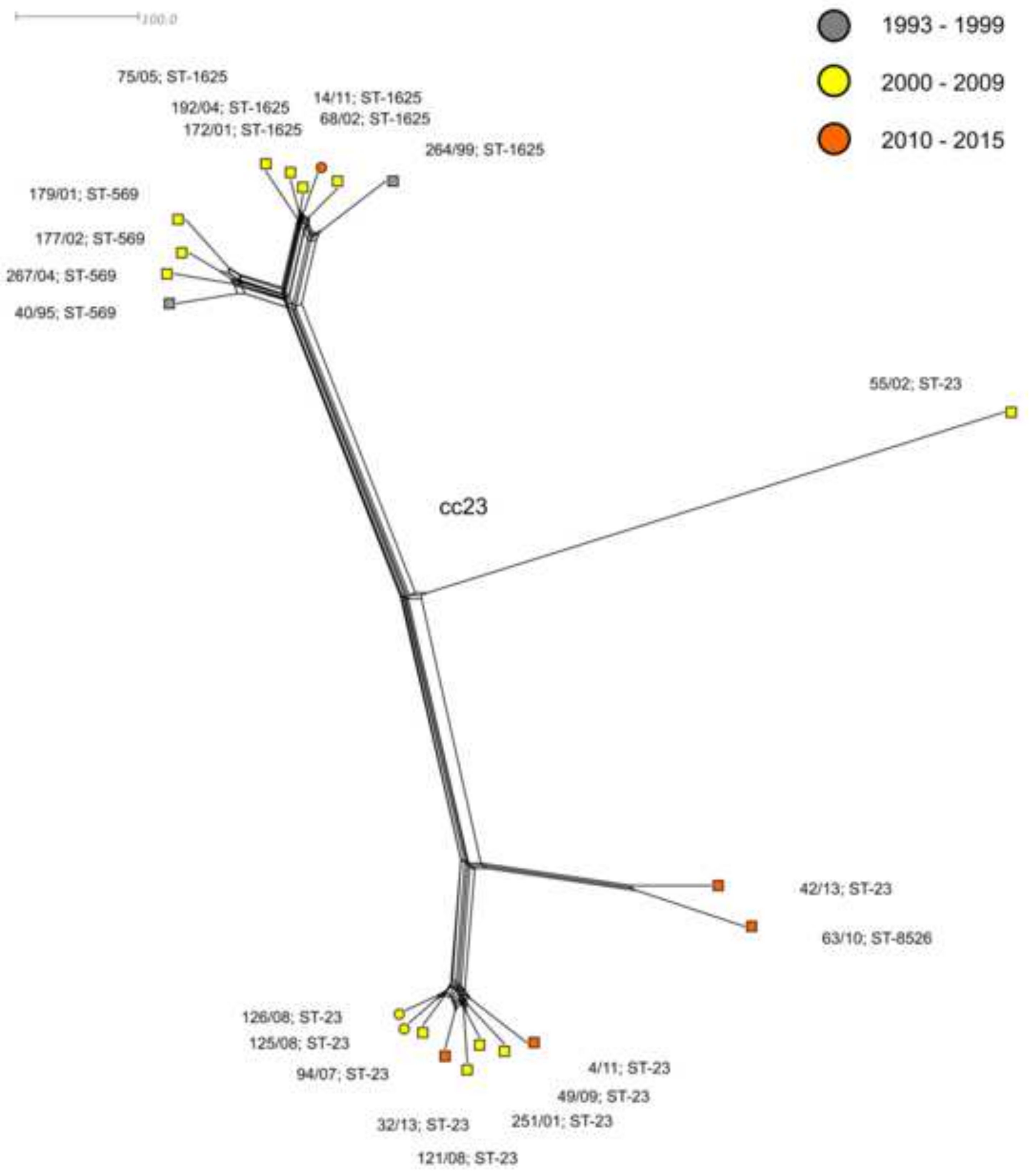

Click here to access/download;Figure;Fig 3A.tif

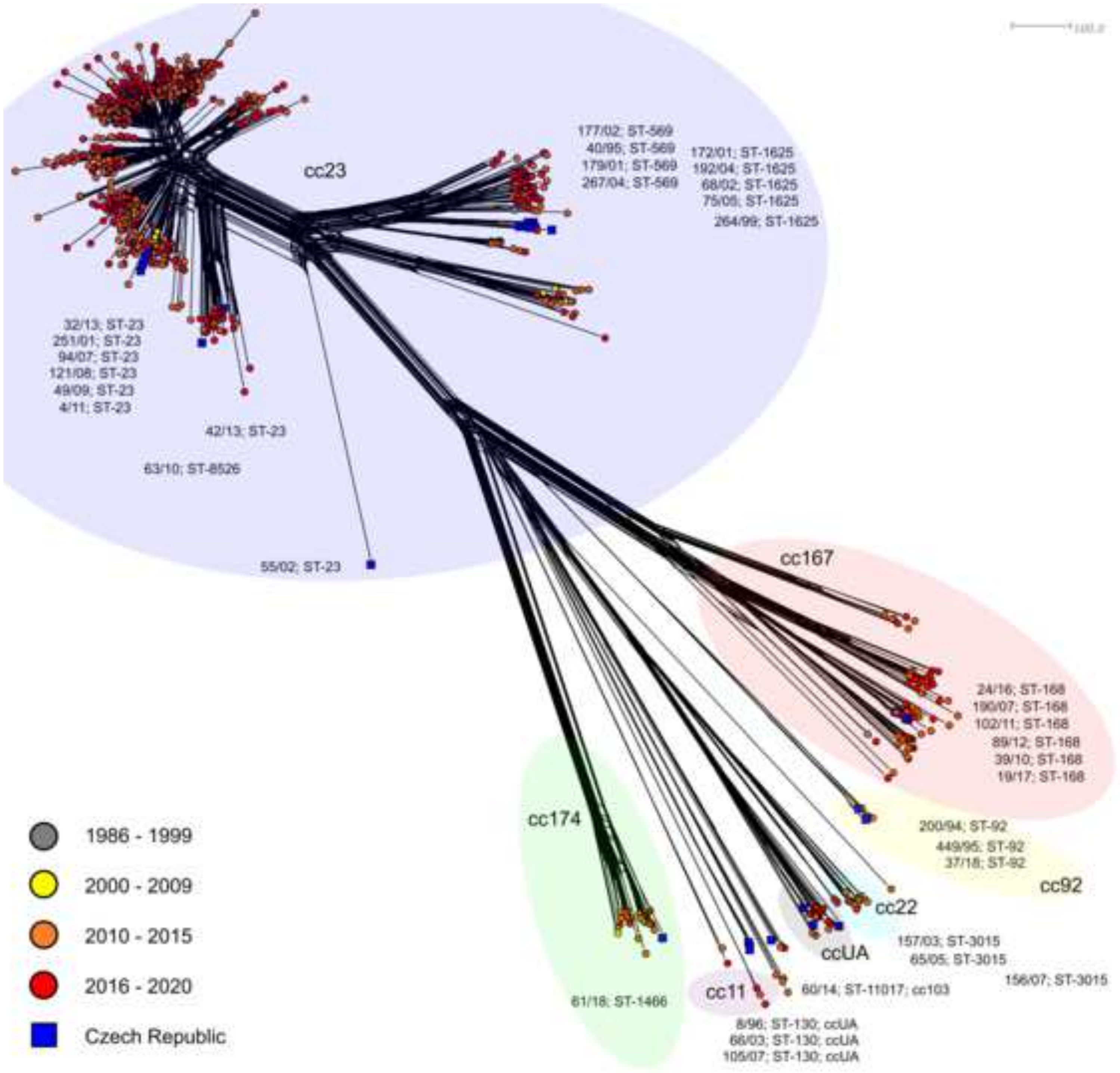

Click here to access/download;Figure;Fig 3B.tif

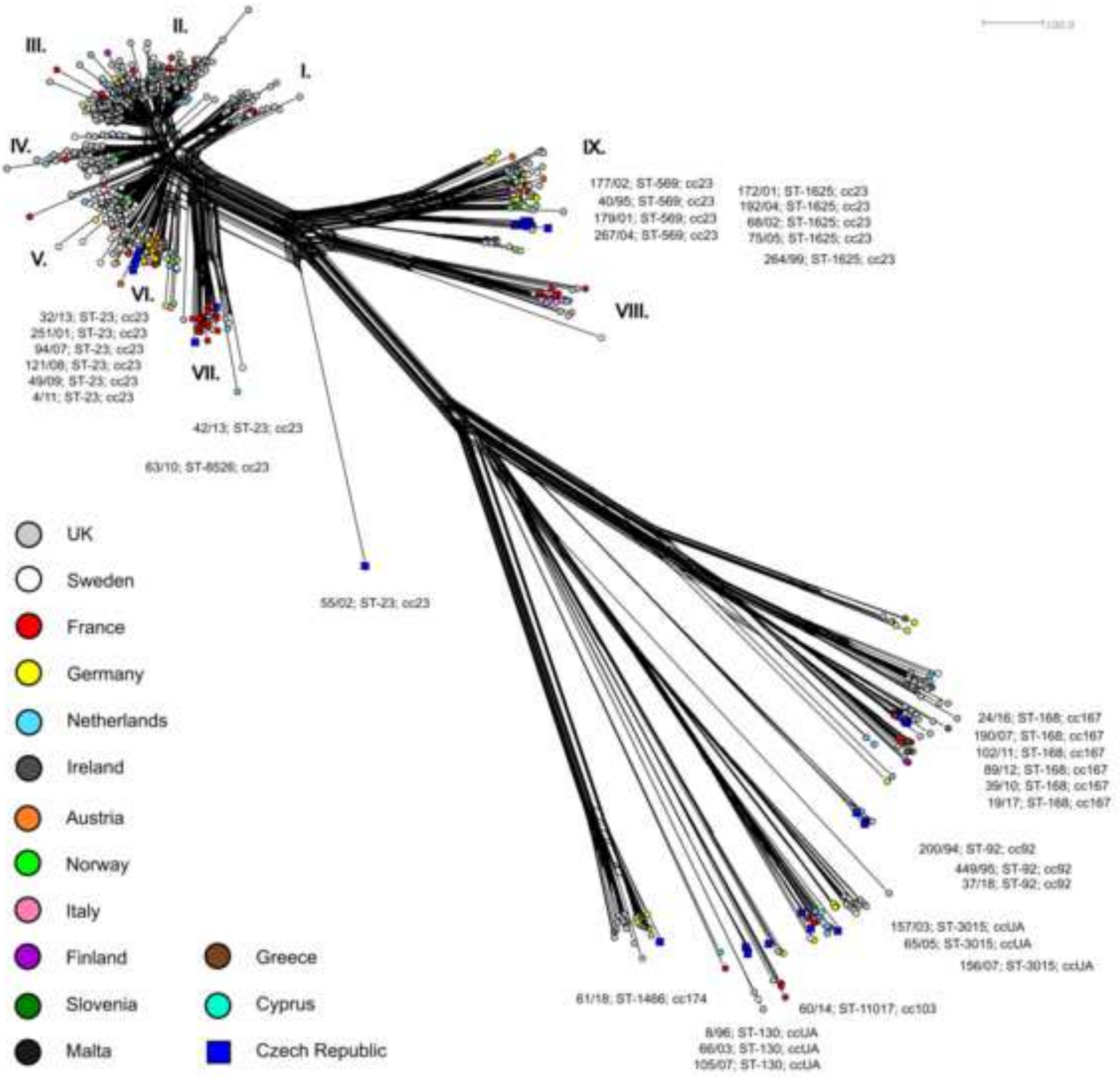

Click here to access/download;Figure;Fig 4A.tif

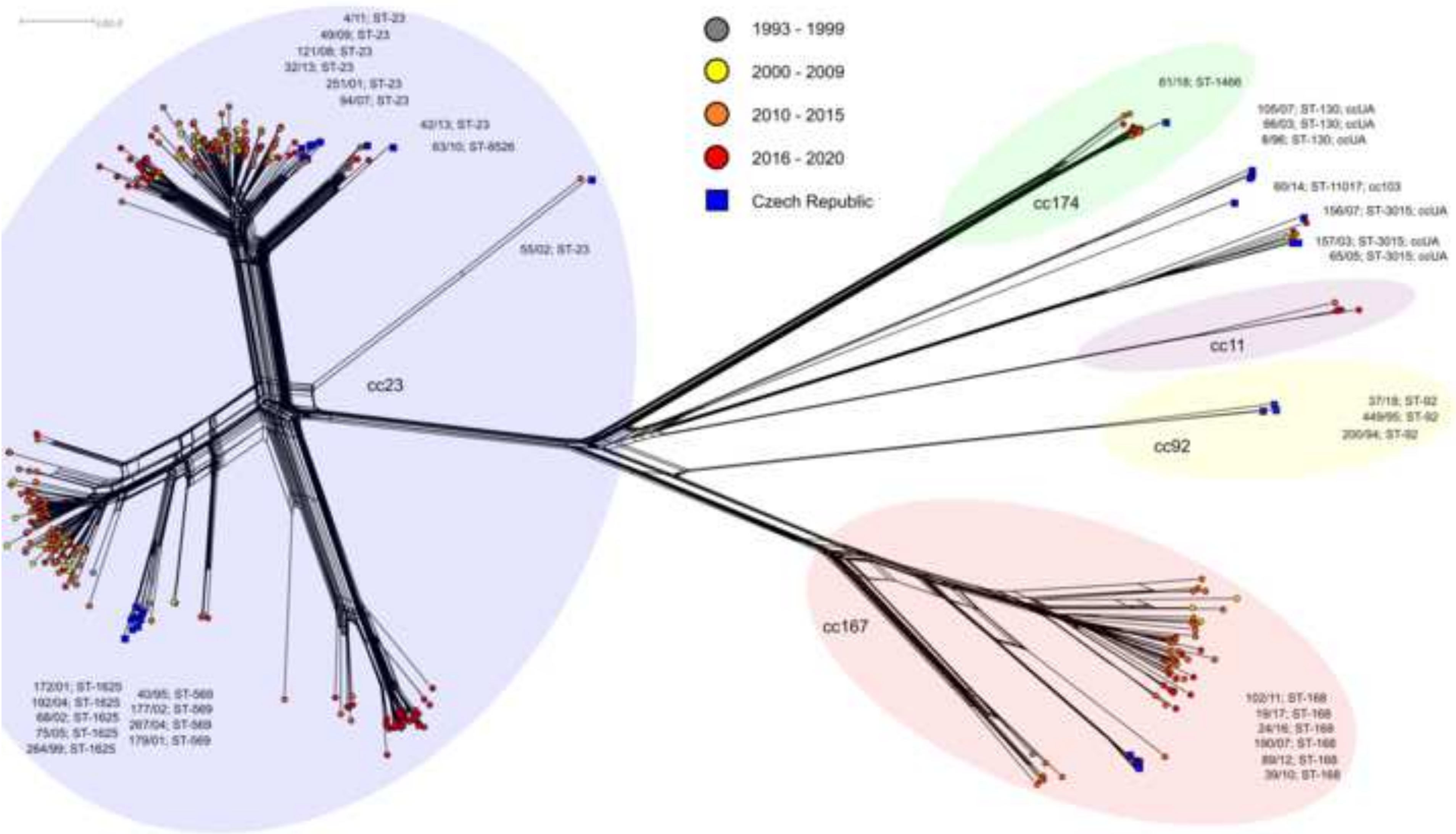

Click here to access/download;Figure;Fig 4B.tif

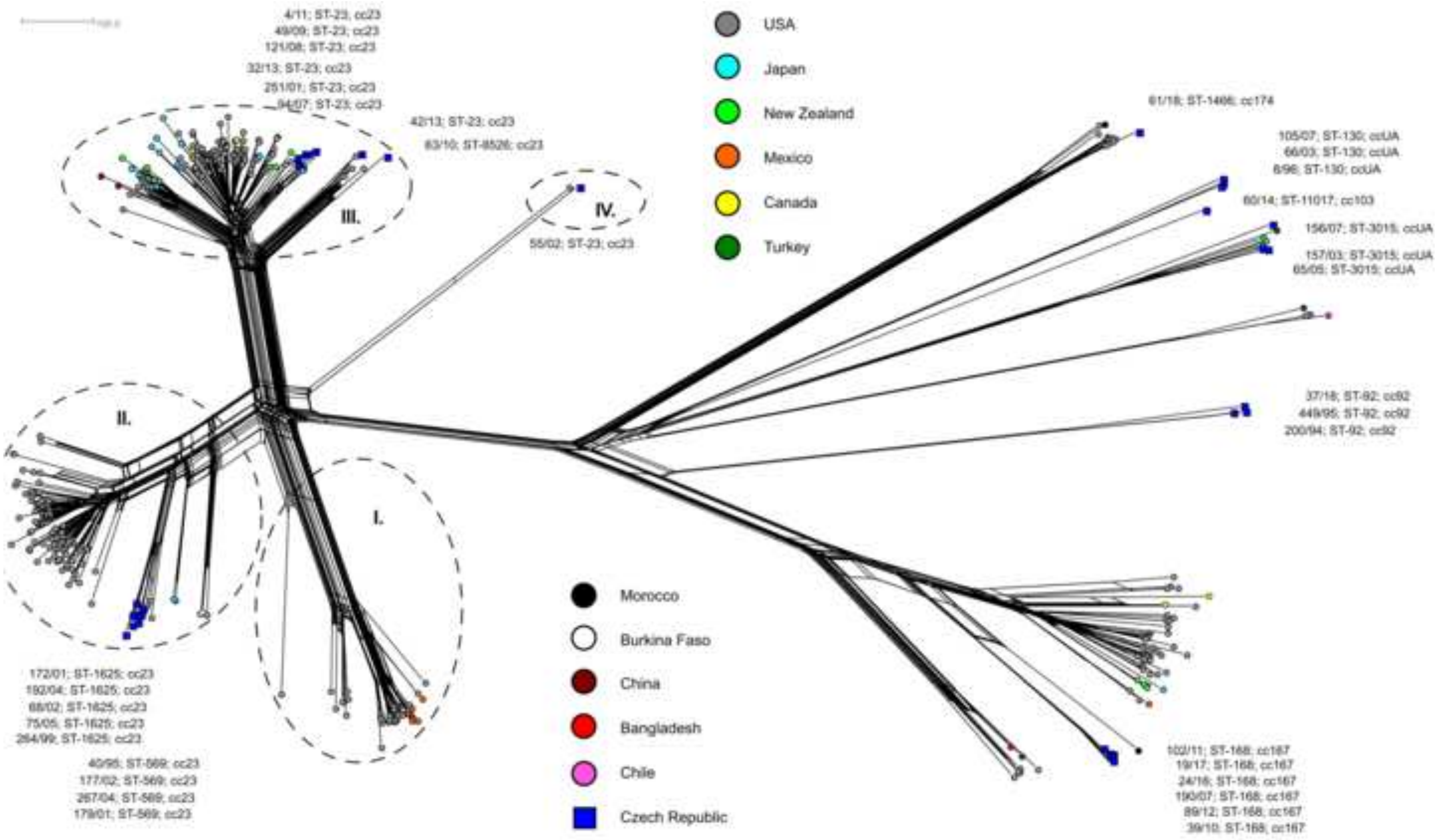

Click here to access/download
Supporting Information
S1 Fig.pdf

Click here to access/download
Supporting Information
S2 Fig.pdf

Click here to access/download
Supporting Information
S3 Fig.pdf
