# Supplementary material for: Whole genome sequencing of Neisseria meningitidis Y isolates collected in the Czech Republic in 1993-2018
Source: PLoS One. 2022 Mar 10;17(3):e0265066. doi: 10.1371/journal.pone.0265066 (PMC8912901; doi:10.1371/journal.pone.0265066)
Supplement: Attachment — Submitted filename: Response to Reviewers.docx [file pone.0265066.s005.docx]

**PONE-D-21-29616**

**Whole Genome Sequencing of Neisseria meningitidis Y isolates collected in the Czech Republic in 1993-2018**

**PLOS ONE**

**The authors thank the academic editor and three reviewers for their valuable comments and recommendations. Most of them were accepted - see the answers to the points below.**

**Response to the academic editor:**

While your paper addresses an interesting question and provide valuable information of the epidemiologically situation of *Neisseria meningitides*, there are significant concern both about the presentation as well as the readability of the manuscript.  In particular, the rationale of the study needs to be strengthen and the aim of the study need to be explained in the abstract and introduction.  The writing was inadequate and accordingly, the data were not following a clear logical line of thought. In addition there were numerous issues identified where additional experimentation and documentation is needed, please see reviewers’ insightful comments below.  On a more personal level, I also have a few issues with the manuscript that needs to be addressed, please see specific comments below.  Furthermore, the quality of the language needs to be improved.  Please have a fluent, preferably native, English-language speaker thoroughly copyedit your manuscript for language usage, spelling, and grammar.  If you do not know anyone who can do this, we suggest you use a professional language editing or copyediting service.

**These recommendations were mostly accepted – please see in the enclosure „Revised Manuscript with Track Changes“.**

Specific comments:

1. Line 49 – 56, combine these two paragraphs into one.

**This recommendation was accepted – please see in the enclosure „Revised Manuscript with Track Changes“.**

2 Line 158, please specify which ribosomal gene?

**This recommendation was accepted – please see in the enclosure „Revised Manuscript with Track Changes“.**

3. Expand the explanation of Table 1 further. For examples, Most prevalent is cc23 with 21 isolates (ST-23, 569,1625, and 8526).  Followed by cc92 (ST-92 and ST-94) with 7 isolates. and cc167 (ST-168) with 6 isolates, as well as one isolate each for cc103 (ST11017) and cc174 (ST1466), etc.

**This recommendation was accepted – please see in the enclosure „Revised Manuscript with Track Changes“.**

4. Table 1 needs substantial editing to provide more relevant information. A few suggestion here:

1) Year of isolation is indicated in the strain ID? Is it necessary to have separate year of isolation?

**This recommendation was accepted – please see in the enclosure „Revised Manuscript with Track Changes“.** 2) Symptoms, you can probably delete this column and put * on the strains ID isolated from carrier and explained in the note; **This recommendation was accepted – please see in the enclosure „Revised Manuscript with Track Changes“.** 3) Delete columns of serogroup and genogroup since all the isolates belong to the same serogroup and genogroup; **This recommendation was accepted – please see in the enclosure „Revised Manuscript with Track Changes“.**

4) Add column to indicate clonal complex, BAST, and rST **The columns indicating clonal complex, BAST and rST were already in Table 1 that was in the first version of the submitted manuscript. Unfortunately, they were not visible. In the revised version of the manuscript which we are now submitting, Table 1 was divided into Table 1 and Table 2 and all columns are visible.**

5. Note for Table 1 needs significant editing to provide relevant information:  1) Unless the authors added column indicating cc, the explanation for cc and UA is unnecessary; **As explained above, unfortunately not all Table 1 columns were visible in the first version of the manuscript.** **In the revised version of the manuscript which we are now submitting, Table 1 was divided into Table 1 and Table 2 and all columns are visible. The explanation for cc and UA is necessary for Table 1.** 2) Line 183 – 185, not sure that these are relevant to the current Table 1. **As explained above, unfortunately not all Table 1 columns were visible in the first version of the manuscript.** **In the revised version of the manuscript which we are now submitting, Table 1 was divided into Table 1 and Table 2 and all columns are visible. This text is relevant for Table 1 and Table 2.**

6. Line 187 – 193, combine these two paragraphs into one.

**This recommendation was accepted – please see in the enclosure „Revised Manuscript with Track Changes“.**

7. Line 307 – 310, are these sentence referring to one of the figures?

**As explained above, unfortunately not all Table 1 columns were visible in the first version of the manuscript.** **In the revised version of the manuscript which we are now submitting, Table 1 was divided into Table 1 and Table 2 and all columns are visible. This text is relevant for Table 2.**

8. Line 314, please explain Trumenba.

**This recommendation was accepted – please see in the enclosure „Revised Manuscript with Track Changes“.**

9. Line 317 – 322, These are confusing sentences. Not sure what the authors wish to convey?

**As explained above, unfortunately not all Table 1 columns were visible in the first version of the manuscript.** **In the revised version of the manuscript which we are now submitting, Table 1 was divided into Table 1 and Table 2 and all columns are visible. This text is relevant for Table 2.**

10. Line 356 – 357, awkward sentence, please rephrase for clarity.

**This recommendation was accepted – please see in the enclosure „Revised Manuscript with Track Changes“.**

11. Discussion needs to focus on discussing the results first and how it compared to other literatures.

**This recommendation was accepted – please see in the enclosure „Revised Manuscript with Track Changes“.**

**Response to the Reviewer #1:**

1- line 33 -37 please rephrase it so it will be more clear
2-In the introduction we have to highlight the importance of our research why did you do sequencing for serogroup Y and not the main groups ,if there were increase in serogroup Y after introducing the MenC and MenB ,now as you mentioned there are 2 quadrivalent vaccines covering the Y serogroup,what is the outcome after doing WGS and the relation to the current vaccine. 3-please try to summarize the description of each figure without going for much details. 4-in the discussion we have to mention our results and compare it with different countries

**These recommendations were accepted – please see in the enclosure „Revised Manuscript with Track Changes“.**

**Response to the Reviewer #2:**

The manuscript entitled “Whole Genome Sequencing of Neisseria meningitidis Y isolates collected in the Czech Republic in 1993-2018” compares meningococcal serogroup Y genomes from the Czech Republic with other European and non-European genomes in order to relate the lineages in the Czech Republic to a global setting. Vaccination against serogroups ACWY and B is recommended in the Czech Republic and the study also includes characterization on theoretical vaccine coverage. Although the results from this study is an important contribution to the long-term epidemiological surveillance of meningococcal isolates in general, and the emergent serogroup Y in particular, the manuscript needs to be clarified and restructured and especially the results and discussion need a major overview according to the points suggested below.
**These recommendations were accepted – please see in the enclosure „Revised Manuscript with Track Changes“.**

**Major points:**

1. The aim of the study is not entirely clear to me and some analyses in this study are incompletely incorporated into the narrative. For example, the theoretical vaccine coverage BAST and MenDeVAR index is not described in M&M and it is not discussed in the discussion section what this information adds. Additionally, although very important, without a clearer aim it is unclear why the Czech isolates were compared to European isolates and then non-European isolates.

**These recommendations were accepted – please see in the enclosure „Revised Manuscript with Track Changes“.**

2. The results section lacks a clear narrative and is difficult to get through. Although there is no limit on word count, the manuscript is 6000 words long and seems to be unbalanced, 3 pages of introduction, 13 pages of results, 3 pages of discussion. The results section would benefit from being shortened and some parts moved to the discussion.
**These recommendations were accepted – please see in the enclosure „Revised Manuscript with Track Changes“.**

3. Figure S1 gives the reader an important sense of how the serogroup Y incidence, although increasing, is still very low in the Czech Republic compared to serogroups B and C. The authors could argue that it is rather the high mortality rate of serogroup Y that is the issue but this needs to be clearer as the introduction focuses on the increased serogroup Y IMD incidence.
**These recommendations were accepted – please see in the enclosure „Revised Manuscript with Track Changes“.**

4. Everything is relative but calling the serogroup Y isolates heterogeneous when 2/3 belonged to cc23 and then looking at non-European isolates which were even more heterogeneous, maybe then one would say that the Czech isolates were quite homogeneous? This could be re-phrased to be a more “soft” statement throughout the manuscript.
**This recommendation was accepted – please see in the enclosure „Revised Manuscript with Track Changes“.**

5. The discussion is incomplete and mostly mentions other studies. The most important findings should be highlighted and how the results adds to the field of research, possible limitations with the study and future directions should be added.
**These recommendations were accepted – please see in the enclosure „Revised Manuscript with Track Changes“.**

6. The manuscript would benefit from a general language revision, especially in regard to grammatical tense.
**This recommendation was accepted – please see in the enclosure „Revised Manuscript with Track Changes“.**

**Minor points:**

All figures would benefit from having the N= written in the figure legends instead of in the main text.

**This recommendation was accepted – please see in the enclosure „Revised Manuscript with Track Changes“.**

Abbreviations NRL, ST and cc in the legends should be explained.
**Abbreviations NRL, ST and cc is explained in Materials and Methods – please see in the enclosure „Revised Manuscript with Track Changes“.**

Line 20-22: The material and methods section of the abstract is somewhat short, it does not for example mention that the Czech isolates were compared to other countries.
**This recommendation was accepted – please see in the enclosure „Revised Manuscript with Track Changes“.**

Line 59: The cause of shifting meningococcal serogroup incidences in meningococci is not completely known and vaccination programmes is probably only part of the explanation, the sentence could be rephrased to e.g. “…partly due to vaccination programmes”.
**This recommendation was accepted – please see in the enclosure „Revised Manuscript with Track Changes“.**

Line 73: Please add a reference to this statement.
**This recommendation was accepted – please see in the enclosure „Revised Manuscript with Track Changes“.**

Line 78: The study was from 2015, not 2014.
**This recommendation was accepted – please see in the enclosure „Revised Manuscript with Track Changes“.**

Line 80: WGS was done in reference number 14.
**This recommendation was accepted – please see in the enclosure „Revised Manuscript with Track Changes“.**

Line 88: What is the timeline for the numbers on serogroup distribution, since 1993?
**This recommendation was accepted – please see in the enclosure „Revised Manuscript with Track Changes“.**

Introduction: please add numbers on IMD incidence in the Czech Republic. These would be interesting to see in conjunction to the vaccine strategy.
**This recommendation was accepted – please see in the enclosure „Revised Manuscript with Track Changes“.**

Line 128: There is no information about the healthy carriers, in which setting were these sampled? What was the purpose of these samples in the study?
**This recommendation was accepted – please see in the enclosure „Revised Manuscript with Track Changes“.**

Material and methods: The BAST, MenDeVAR index and rMLST are not mentioned.
**This recommendation was accepted – please see in the enclosure „Revised Manuscript with Track Changes“.**

Table 1: The entire table is not visible. The abbreviations “CAR” and “PAC” are not explained.
**As explained above, unfortunately not all Table 1 columns were visible in the first version of the manuscript.** **In the revised version of the manuscript which we are now submitting, Table 1 was divided into Table 1 and Table 2 and all columns are visible. The column with the abbreviations CAR and PAC was deleted according to the recommendation of the academic editor. Isolates from IMD versus isolates from carriers are distinguished by bold in the column Strain IDs in Table 1 and Table 2.**

Line 215-216: Please give an estimation on how many the minimal genetic differences are.
**This comment was accepted, the part of sentence about minimal genetic differences was deleted – please see in the enclosure „Revised Manuscript with Track Changes“.**

Line 295-303: This should be mentioned in the Material and Methods section.
**This recommendation was accepted – please see in the enclosure „Revised Manuscript with Track Changes“.**

Line 479: Please use “n=3” instead of “3x ST-2880”, should be changed throughout the manuscript.
**The expressions such as "3x" have been deleted from the text throughout the manuscript as part of the recommended shortening of the text.**

Line 494-503: This type of reasoning is better fitted for the discussion section rather than the results.
**This recommendation was accepted – please see in the enclosure „Revised Manuscript with Track Changes“.**

Line 515: The discussion starts with comparisons of which STs dominates in different countries, suggestion to add that the YI strain in Sweden belongs to ST-23.
**This recommendation was accepted – please see in the enclosure „Revised Manuscript with Track Changes“.**

Discussion: In the introduction it is stated that molecular characterization of serogroup Y provides background data for vaccination strategy, how the results from the current study will impact vaccination strategies could be discussed.
**This recommendation was accepted – please see in the enclosure „Revised Manuscript with Track Changes“.**

Fig 3-4: The roman letters are not explained in the figure legend.

**This recommendation was accepted – please see in the enclosure „Revised Manuscript with Track Changes“.**

**Response to the Reviewer #3:**

Your work represents a nice work and adds valuable information of the epidemiologically situation based on whole genome sequencing of Neisseria meningitides serogroup Y isolates, collected in the Czech Republic during 1993-2018, and also its relation to other international isolates. The manuscript though needs extensive revisions, especially regarding the structure and focus of the scope of aim, i.e all parts regarding vaccination need to be shortened or in some places even taken away.

**These recommendations were accepted – please see in the enclosure „Revised Manuscript with Track Changes“.**

You can see more in detail below.

1. A specified aim is missing both in the manuscript and in the abstract.
**This recommendation was accepted – please see in the enclosure „Revised Manuscript with Track Changes“.**

2. The introduction is too long and need to be both structured and written in a logic way. For example, the section about vaccination is to detailed and needs to be shortened substantially, it should also be mentioned that the incidence and variation of causative serogroups varies over time not only due to vaccination but also natural fluctuations.
**These recommendations were accepted – please see in the enclosure „Revised Manuscript with Track Changes“.**

3. In M&M the collection of carrier isolates need to be described how it was done.
**This recommendation was accepted – please see in the enclosure „Revised Manuscript with Track Changes“.**

4. All subheadings (in the entire manuscript) need to be optimised and shortened, i.e “DNA extraction, WGS protocol, and WGS data analysis and visualization” could be Whole genome sequencing and analysis or even divided into sections.
**This recommendation was accepted – please see in the enclosure „Revised Manuscript with Track Changes“.**

5. The technically sound of the sequencing must be improved.
**We do not understand this recommendation. The sequencing used in the present study have been described in detail previously, as is mentioned in M&M part [ref. 17 and 18].**

6. All abbreviations in the Tables and figures need to be explained in footnotes or used without abbreviation and print full name, especially in the headings (should be possible to read stand-alone).
**This recommendation was accepted – please see in the enclosure „Revised Manuscript with Track Changes“.**

7. The result section is too long and should only include the actual results and not new background information or interpretations. Do not repeat all results that can be found in tables and figures, just the most important findings.
**This recommendation was accepted – please see in the enclosure „Revised Manuscript with Track Changes“.**

8. The interpretations can be moved to the section of discussion. Also do not include all results from every country in the discussion only the comparison.
**This recommendation was accepted – please see in the enclosure „Revised Manuscript with Track Changes“.**

9. The conclusion (both in manuscript and abstract) needs to answer an aim (that first has to be defined…).
**This recommendation was accepted – please see in the enclosure „Revised Manuscript with Track Changes“.**

10. All figures need better solution.

**When submitting the corrected version of the manuscript, we have uploaded our figure files to the Preflight Analysis and Conversion Engine (PACE) digital diagnostic tool   https://pacev2.apexcovantage.com/ to be sure that our figures meet PLOS requirements.**
