# Supplementary material for: Whole genome sequencing of Neisseria meningitidis Y isolates collected in the Czech Republic in 1993-2018
Source: PLoS One. 2022 Mar 10;17(3):e0265066. doi: 10.1371/journal.pone.0265066 (PMC8912901; doi:10.1371/journal.pone.0265066)
Supplement: Attachment — Submitted filename: Recommendations for manuscript.docx [file pone.0265066.s006.docx]

Recommendations for manuscript titled : **Whole Genome genome** **Sequencing sequencing of *Neisseria meningitidis* Y isolates collected in the Czech Republic in 1993-2018**

General overview :

I want to thank the authors for the interesting research and for the meticulous detailed points mentioned in the manuscript.I have few points some are recommendations and others are inquiries .

This manuscript has been re-written and reads much better as suggested by the previous reviewers . However further editing for clarity would be advised.

Abstract:

Line 38-39: The WGS has been described previously (This Phrase can be removed and mentioned in the M&M)

Any mention about studying the relatedness to vaccine strains in the abstract ?whether in the aim ,results ?

Introduction:

Line 105-109

The Swedish isolates comment can be removed from the introduction as you already mentioned it in the discussion

Methods

Paragraph from line 208 to 226 : would you clarify why you choose Vaccine covering group B ,you mentioned there are some protection but why we are studying vaccine covering group B and you have already vaccine covering Y strains why these vaccines were not studied ? would you elaborate more in this point .

Results:

Paragraph starting by line 269,these results of similarities between vaccines for Men B and the studied isolates, what is the interpretation of these results ,are you going to recommend these vaccines instead of the current vaccines for Y strains ?what is your recommendation after mentioning the color coding similarities

Is it possible to mention this in the discussion part and how these results will update the vaccine strategy .

Line 347: would you mention at the beginning how many invasive and how many carriers having the CC92 and the same for CC23

Too much details for the infrequent CC ,better to summarize this part only mentioning the main findings.

Fig3A and 3B have the same title ?

Paragraph started by line 495: Too much details ,you can summarize this paragraph as this is the infrequent non CC23,you can put all non 23CC in one paragraph.

Paragraph started by line 567: summarize lineage ii the main findings you cannot describe each and every sub-lineage ,only general description because everything is shown in the figure so you mention what you cannot describe in the figure

Discussion :

Line 648 : I think this was mentioned before by one of the reviewer that if 2/3 of isolates are CC23 how can we considered that as heterogeneous?

Line657: The non-European population of isolates of N. meningitidis Y appears to be less diverse or more diverse

Line 670 to 679 would you rephrase the sentence please, the meaning is not clear (the whole paragraph need to be rewritten )

Line 744 to 748: how these results will update on the strategy of vaccine coverage in Czech Republic.
