# Supplementary material for: Whole genome sequencing of Neisseria meningitidis Y isolates collected in the Czech Republic in 1993-2018
Source: PLoS One. 2022 Mar 10;17(3):e0265066. doi: 10.1371/journal.pone.0265066 (PMC8912901; doi:10.1371/journal.pone.0265066)
Supplement: Attachment — Submitted filename: Response to Reviewers.docx [file pone.0265066.s007.docx]

**PONE-D-21-29616R1**

**Whole genome sequencing of *Neisseria meningitidis* Y isolates collected in the Czech Republic in 1993-2018**

**PLOS ONE**

**The authors thank the academic editor and two reviewers for their valuable comments and recommendations. Most of them were accepted - see the answers to the points below.**

**Response to the academic editor:**

Both reviewers agreed that the revised manuscript showed substantial improvement, however, some new points have arisen and need to be addressed carefully.  Please see reviewers’ insightful comments below.  Also, there are numerous issues where additional clarification is needed (specific comments).

**These recommendations were mostly accepted – please see in the enclosure „Revised Manuscript with Track Changes“.**

In addition, the quality of the language still needs to be improved.  We suggest you thoroughly copyedit your manuscript for language usage, spelling, and grammar. If you do not know anyone who can help you do this, you may wish to consider employing a professional scientific editing service.

**The English proofing of the second revised version of the manuscript was carried out by Keith Jolley of the University of Oxford, who, in addition to being a native speaker, is also a world-renowned expert in whole genome sequencing of *Neisseria meningitidis*.**

Specific comments:

1. Line 193 – 195:  The statement “Analysis of the theoretical…” is not relevant to the manuscript, suggest deletion.

**This recommendation was accepted – please see in the enclosure „Revised Manuscript with Track Changes“.**

2. Line 215:  Suggest adding the following statement "The highest incidence of IMD caused by *N. meningitidis* Y in Czech Republic is in adolescents/young adults  (age 15 - 19), followed by older adults (≥65 years of age) and in younger children (1 - 4 years old). (Fig. S3)”  Suggest changing to Fig. S3 to S2 in order to reflect the fact that Fig. S3 appeared first.

**This recommendation was accepted – please see in the enclosure „Revised Manuscript with Track Changes“.**

3. Line 249:  Suggest adding the following statement “The highest incidence of IMD caused by *N. meningitidis* Y in Czech Republic is in adolescents/young adults  (age 15 - 19), followed by older adults (≥65 years of age) and in younger children (1 - 4 years old)”

**This information would already be duplicated, given that we have already added the age information above, based on a previous recommendation.**

4. Line 277:  Are you referred to fig.2?  If so, please mentioned here.

**This recommendation was accepted – please see in the enclosure „Revised Manuscript with Track Changes“.**

5. Line 277:  Suggest changing “A more detailed representation shows…” to “A more detailed phylogenetic analysis of cc23 isolates (Fig. 2) showed…”

**This recommendation was accepted – please see in the enclosure „Revised Manuscript with Track Changes“.**

6. Line 334:  It will also be nice to break out how many isolates are from 1986 - 1999, 2000 - 2009, 2010 - 2015, 2016 – 2020.

**This recommendation was accepted – please see in the enclosure „Revised Manuscript with Track Changes“.**

7. Line 336 – 343:  Combine Fig. 3A and 3B legend into one.  **Fig 3. Genetic diversity of *N. meningitidis* Y invasive isolates from the Czech Republic and European countries.** A) Genetic diversity of *N. meningitidis* Y isolates collected from 1986 to 2020:  Isolates (n = 995) are coloured according to detection year. Invasive isolates from Czech Republic (n = 35) are marked with a dark blue square and labelled by their NRL number, ST, and cc. B) Genetic relationship of *N. meningitidis* Y isolates based on geographic origins:  Isolates (n = 995) are coloured according to the country of origin. The individual lineages of the cc23 clonal complex are marked by Roman numerals (I – IX).  In addition, suggest including similar coloring scheme for Fig. 3B.

**This recommendation was accepted – please see in the enclosure „Revised Manuscript with Track Changes“. We do not understand the additional suggestion to include similar colors in Fig. 3B, because Fig. 3A presents different periods while Fig. 3B presents different countries.**

8. Line 352 – 354:  Fig. 3A seems to show that cc23 were more prevalent/diversified from 2010 - 2020.  Only one from 1986 - 1999 and few from 2000 - 2010 which does not collaborate the statement here. Please clarify.

**This recommendation was accepted – please see in the enclosure „Revised Manuscript with Track Changes“.**

9. Line 354:  Change “…all time period” to “…all time period (Fig. 3B)”.  Also suggest using similar coloring scheme for Fig. 3B.

**This recommendation was accepted – please see in the enclosure „Revised Manuscript with Track Changes“. We do not understand the additional suggestion to include similar colors in Fig. 3B, because Fig. 3A presents different periods while Fig. 3B presents different countries.**

10. Line 402:  Similarly, it will also be nice to break out how many isolates are from 1993 - 1999, 2000 - 2009, 2010 - 2015, 2016 – 2020.

**This recommendation was accepted – please see in the enclosure „Revised Manuscript with Track Changes“.**

11. Line 404 – 413:  Similarly, please combine Fig. 4A and 4B legend into one. **Fig 4. Genetic diversity of *N. meningitidis* Y invasive isolates from the Czech Republic** **and non-European countries.** A) Genetic diversity of *N. meningitidis* Y isolates collected from 1993 to 2020:  Isolates (n = 410) are coloured according to detection year. Invasive isolates from Czech Republic (n = 35) are marked with a dark blue square and labelled by their NRL number, ST, and cc. B) Genetic relationship of *N. meningitidis* Y isolates based on geographic origins:  Isolates (n = 410) are coloured according to the country of origin. The individual lineages of the cc23 clonal complex are marked by Roman numerals (I – IV). Invasive isolates from Czech Republic (n =35) are marked with a dark blue square and labelled by their NRL number, ST, and cc.  Also, Fig.4B should retain the same coloring as Fig. 4A as well.

**This recommendation was accepted – please see in the enclosure „Revised Manuscript with Track Changes“. We do not understand the additional suggestion to include similar colors in Fig. 4B, because Fig. 4A presents different periods while Fig. 4B presents different countries.**

12. Line 514:  Why discuss the cc23 in Sweden?  Does this correlate with Czech?

**This recommendation was accepted – please see in the enclosure „Revised Manuscript with Track Changes“.**

13. Discussion:  Length discussion of cc23 in Sweden, England, Wales, USA, and Canada. Not sure what the authors wish to convey.  Suggest consolidating these sections and shorten the discussion.

**This recommendation was accepted – please see in the enclosure „Revised Manuscript with Track Changes“.**

**Response to the Reviewer #1:**

**Abstract:**
Line 38-39: The WGS has been described previously (This Phrase can be removed and mentioned in the M&M)

Any mention about studying the relatedness to vaccine strains in the abstract ?whether in the aim ,results ?

**This recommendation was accepted – please see in the enclosure „Revised Manuscript with Track Changes“.**

**Introduction:**
Line 105-109
The Swedish isolates comment can be removed from the introduction as you already mentioned it in the discussion

**This recommendation was accepted – please see in the enclosure „Revised Manuscript with Track Changes“.**

**Methods**
Paragraph from line 208 to 226 : would you clarify why you choose Vaccine covering group B ,you mentioned there are some protection but why we are studying vaccine covering group B and you have already vaccine covering Y strains why these vaccines were not studied ? would you elaborate more in this point .

**This recommendation was accepted – please see in the enclosure „Revised Manuscript with Track Changes“.**

**Results:**
Paragraph starting by line 269,these results of similarities between vaccines for Men B and the studied isolates, what is the interpretation of these results ,are you going to recommend these vaccines instead of the current vaccines for Y strains ? what is your recommendation after mentioning the color coding similarities
**This recommendation was accepted – please see in the enclosure „Revised Manuscript with Track Changes“.**

Is it possible to mention this in the discussion part and how these results will update the vaccine strategy .
**This was already stated in the previous version of the manuscript: …**in the Czech Republic the conjugated tetravaccine ACYW started to be used instead of the previous conjugated monovaccine C. **- so we leave it unchanged.**

Line 347: would you mention at the beginning how many invasive and how many carriers having the CC92 and the same for CC23
**This was already stated in the previous version for cc92 - so we are adding it also for cc23 – please see in the enclosure „Revised Manuscript with Track Changes“.**

Too much details for the infrequent CC ,better to summarize this part only mentioning the main findings.
**This recommendation was accepted – please see in the enclosure „Revised Manuscript with Track Changes“.**

Fig3A and 3B have the same title ?
**This recommendation is similar to the recommendation of the academic editor (see above) and was accepted: the title of Fig. 3A and 3B has been merged together – please see in the enclosure „Revised Manuscript with Track Changes“.**

Paragraph started by line 495: Too much details ,you can summarize this paragraph as this is the infrequent non CC23,you can put all non 23CC in one paragraph.
**This recommendation was accepted – please see in the enclosure „Revised Manuscript with Track Changes“.**

Paragraph started by line 567: summarize lineage ii the main findings you cannot describe each and every sub-lineage ,only general description because everything is shown in the figure so you mention what you cannot describe in the figure
**This recommendation was accepted – please see in the enclosure „Revised Manuscript with Track Changes“.**

**Discussion :**
Line 648 : I think this was mentioned before by one of the reviewer that if 2/3 of isolates are CC23 how can we considered that as heterogeneous?

**This recommendation was accepted – please see in the enclosure „Revised Manuscript with Track Changes“.**

Line657: The non-European population of isolates of N. meningitidis Y appears to be less diverse or more diverse

**It was already stated in the previous version of the manuscript that the non-European population appears to be less diverse - so we leave it unchanged.**

Line 670 to 679 would you rephrase the sentence please, the meaning is not clear (the whole paragraph need to be rewritten )

**This recommendation was accepted – please see in the enclosure „Revised Manuscript with Track Changes“.**

Line 744 to 748: how these results will update on the strategy of vaccine coverage in Czech Republic.

**This was already stated in the previous version of the manuscript: …**in the Czech Republic the conjugated tetravaccine ACYW started to be used instead of the previous conjugated monovaccine C. **- so we leave it unchanged.**

**Response to the Reviewer #2:**

Lines 218-223, it is a bit confusing listing the colors according to MenDeVar index here without a reference to a table. The colors could be left out in the material and method section and clarified in the table instead.
**This recommendation was accepted – please see in the enclosure „Revised Manuscript with Track Changes“.**

Line 723-724, it is unclear why cc175 is mentioned in this paragraph, is this not related to the previous paragraph and should be moved to line 717?

**This recommendation was accepted – please see in the enclosure „Revised Manuscript with Track Changes“.**

January 20, 2022	Pavla Krizova, M.D., Ph.D. – corresponding author
